# Scalable production of tissue-like vascularized liver organoids from human PSCs

**DOI:** 10.1038/s12276-023-01074-1

**Published:** 2023-09-01

**Authors:** Sean P. Harrison, Richard Siller, Yoshiaki Tanaka, Maria Eugenia Chollet, María Eugenia de la Morena-Barrio, Yangfei Xiang, Benjamin Patterson, Elisabeth Andersen, Carlos Bravo-Pérez, Henning Kempf, Kathrine S. Åsrud, Oleg Lunov, Alexandr Dejneka, Marie-Christine Mowinckel, Benedicte Stavik, Per Morten Sandset, Espen Melum, Saphira Baumgarten, Flavio Bonanini, Dorota Kurek, Santosh Mathapati, Runar Almaas, Kulbhushan Sharma, Steven R. Wilson, Frøydis S. Skottvoll, Ida C. Boger, Inger Lise Bogen, Tuula A. Nyman, Jun Jie Wu, Ales Bezrouk, Dana Cizkova, Javier Corral, Jaroslav Mokry, Robert Zweigerdt, In-Hyun Park, Gareth J. Sullivan

**Affiliations:** 1 Hybrid Technology Hub-Centre of Excellence, Institute of Basic Medical Sciences, University of Oslo, Oslo, Norway; 2 Department of Pediatric Research, Oslo University Hospital, Oslo, Norway; 3 Department of Molecular Medicine, Institute of Basic Medical Sciences, University of Oslo, Oslo, Norway; 4 Department of Genetics, Yale Stem Cell Center, Child Study Center, Yale School of Medicine, New Haven, USA; 5 Department of Medicine, Faculty of Medicine, Maisonneuve-Rosemont Hospital Research Center (CRHMR), University of Montreal, Montreal, Canada; 6 Research Institute of Internal Medicine, Oslo University Hospital, Oslo, Norway; 7 Department of Haematology, Oslo University Hospital, Oslo, Norway; 8 Servicio de Hematología y Oncología Médica, Hospital Universitario Morales Meseguer, Centro Regional de Hemodonación, Universidad de Murcia, IMIB, CIBERER, Murcia, Spain; 9 Department: Leibniz Research Laboratories for Biotechnology and Artificial Organs (LEBAO), Hannover Medical School, Hannover, Germany; 10 Norwegian PSC Research Center, Department of Transplantation Medicine, Oslo University Hospital, Oslo, Norway; 11 Institute of Clinical Medicine, Faculty of Medicine, University of Oslo, Oslo, Norway; 12 Department of Optical and Biophysical Systems, Institute of Physics of the Czech Academy of Sciences, Prague, Czech Republic; 13 Section for Gastroenterology, Department of Transplantation Medicine, Oslo University Hospital, Oslo, Norway; 14 European Reference Network RARE-LIVER, Hamburg, Germany; 15 Mimetas, Leiden, The Netherlands; 16 Department of Chemistry, University of Oslo, P.O. Box 1033, Blindern, NO-0315 Oslo, Norway; 17 Department of Forensic Sciences, Oslo University Hospital, Oslo, Norway; 18 Department of Immunology, University of Oslo and Oslo University Hospital, Oslo, Norway; 19 Department of Engineering, Faculty of Science, Durham University, Durham, DH1 3LE United Kingdom; 20 Department of Medical Biophysics, Faculty of Medicine in Hradec Králové, Charles University, Hradec Králové, Czech Republic; 21 Department of Histology and Embryology, Faculty of Medicine in Hradec Králové, Charles University, Hradec Králové, Czech Republic

**Keywords:** Gastrointestinal models, Stem-cell differentiation

## Abstract

The lack of physiological parity between 2D cell culture and in vivo culture has led to the development of more organotypic models, such as organoids. Organoid models have been developed for a number of tissues, including the liver. Current organoid protocols are characterized by a reliance on extracellular matrices (ECMs), patterning in 2D culture, costly growth factors and a lack of cellular diversity, structure, and organization. Current hepatic organoid models are generally simplistic and composed of hepatocytes or cholangiocytes, rendering them less physiologically relevant compared to native tissue. We have developed an approach that does not require 2D patterning, is ECM independent, and employs small molecules to mimic embryonic liver development that produces large quantities of liver-like organoids. Using single-cell RNA sequencing and immunofluorescence, we demonstrate a liver-like cellular repertoire, a higher order cellular complexity, presenting with vascular luminal structures, and a population of resident macrophages: Kupffer cells. The organoids exhibit key liver functions, including drug metabolism, serum protein production, urea synthesis and coagulation factor production, with preserved post-translational modifications such as N-glycosylation and functionality. The organoids can be transplanted and maintained long term in mice producing human albumin. The organoids exhibit a complex cellular repertoire reflective of the organ and have de novo vascularization and liver-like function. These characteristics are a prerequisite for many applications from cellular therapy, tissue engineering, drug toxicity assessment, and disease modeling to basic developmental biology.

## Liver model: New 3D approach yields powerful tool

A unique 3D approach results in an organoid, a laboratory-grown miniature organ model, with high cellular complexity that closely mimics the three-dimensional structure and functions of the liver. The Sullivan lab at Oslo University Hospital, Norway, and co-workers have developed an approach to create liver organoids that results in cellular diversity and is not reliant on costly growth factors or extracellular matrices . They used suspension culture system, in which human pluripotent stem cells self-aggregate into 3D structures, rather than relying on the 2D patterning. Their organoids demonstrated mature cellular behaviours, included a population of liver-specific macrophages, and is capable of drug metabolism, protein production and other key liver functions. The team’s approach is scalable, inexpensive and has multiple potential applications, from basic biology and disease modelling to tissue engineering and cellular therapies.

## Introduction

The liver is the largest endocrine organ in the body and is critical for maintaining homeostasis, serving as the primary site of xenobiotic metabolism, production of coagulation factors, and removal of ammonia as well as a multitude of other essential functions^1^. For liver failure, no other curative treatment approach besides liver transplantation (LTX) exists. Additionally, the gold standard for the evaluation of hepatic metabolism and drug toxicity, among other things, is primary human hepatocytes (PHHs). However, PHHs are extremely limited in supply and rapidly lose function in vitro. This issue indicates the need to identify potential surrogates to fill this void, providing a stable, scalable and genetically defined alternative.

Human pluripotent stem cells (hPSCs) can self-organize into organotypic structures called organoids. However, organoid models do not fully recapitulate the cellular diversity and architectural features of the organ in question^2^. Along with this limitation, current approaches are labor intensive, highly dependent on expensive growth factors and not amenable to scaling. A lack of organotypic in vitro models has led us to develop an approach that utilizes a suspension culture system, leveraging off the ability of hPSCs to self-aggregate upon seeding as single cells. We have overcome the requirement to pattern hPSCs in a 2D format to definitive endoderm^3^ by directly patterning in 3D pluripotent aggregates in suspension in an extracellular matrix (ECM)- and growth factor-independent manner. Additionally, this approach is scalable to generate mini-liver organoids that contain vasculature while recapitulating the complexity of cell types associated with the liver. The resulting organoids also replicate functional features of the liver, including cytochrome p450 activity, which is long lived, and other phase 1 metabolizing enzymes, such as carboxyl esterases, which are involved in xenobiotic metabolism, as well as the ability to synthesize urea. We demonstrate that the organoids have a scavenger function via uptake of labeled substrates. We also demonstrate the utility of this platform to allow modeling of biological processes and potentially disease. An area of particular interest is the coagulation machinery. The organoids produce and secrete a myriad of functional liver-specific proteins, including albumin and serpins (alpha-1 antitrypsin (A1AT) and antithrombin (AT), among others). Additionally, coagulation factors have post-translational modifications (PTMs) and are produced at levels comparable to those in primary hepatocytes. We demonstrate that the coagulation machinery is functional with respect to Factor VII and will provide an important model to study coagulation, characterize coagulation factor deficiencies, and set the basis for the development of cell-based therapy.

## Materials and Methods

### hPSC Culture

The hPSC lines utilized in this study were as follows: the human embryonic stem cell lines H1 (WiCell) and 207^4^ and the previously described human induced pluripotent stem cell (hiPSC) lines AG27 (reprogrammed using retrovirus (Vectalys) from AG05836B fibroblasts, obtained from Coriell Cell Repositories)^5–8^. hPSCs were maintained under feeder-free conditions on Geltrex (Life Technologies)-coated tissue culture plates using Essential 8 medium made in house as described previously^8^.

### Culture of primary human hepatocytes

Human plateable hepatocytes (primary hepatocytes (PHs)) were purchased from Thermo Fisher Scientific and cultured in Williams’ Medium E (1x, no phenol red) (Thermo Fisher Scientific) following the manufacturer’s instructions.

### Two-dimensional hepatocyte-like-cell differentiation

Cells were differentiated into hepatocyte-like cells (HLCs) in 2D as described previously^5,6,8^. Briefly, cells were initially seeded onto Geltrex-coated tissue culture plates as single cells after incubation with Accutase (Life Technologies). The optimal seeding density was previously established empirically as described in Mathapati et al.^5^. Differentiation to HLCs was a 3-stage process consisting of differentiation to definitive endoderm (DE—Phase I), hepatic specification to hepatoblasts/hepatic endoderm (HE—Phase II), and finally maturation to hepatocyte-like cells (HLCs—Phase III). For a detailed protocol of the differentiation process, please see Mathapati et al., 2016 and Siller et al.^5,8^.

### Suspension culture and differentiation into liver organoids

**Fig. 1:**
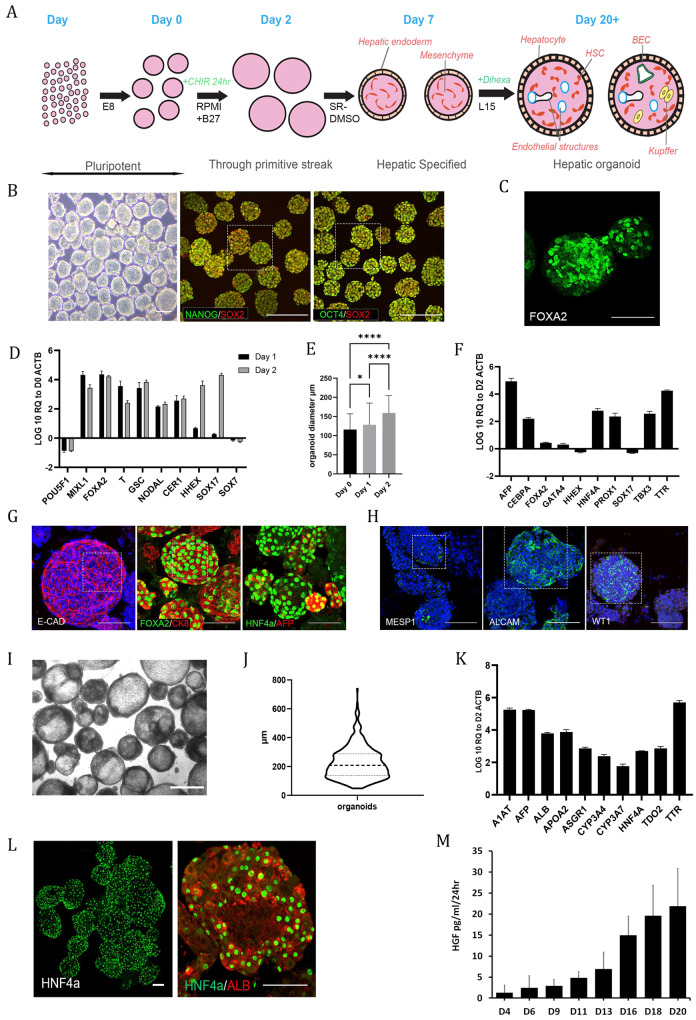
Differentiation of multicellular liver organoids from human PSCs mimics stages of in vivo development. **A** Schematic overview of organoid differentiation from PSCs. **B** Representative images of Day 0 (D0) pluripotent spheroids, left panel brightfield. The remaining panels show whole-mount immunostaining of the pluripotency markers OCT4, SOX2 and NANOG; the scale bar is 200 µm. The highlighted area is magnified in Supplementary Fig. 9. **C** Whole-mount immunostaining of the definitive endoderm (DE) marker FOXA2 at D2 of differentiation. **D** RT‒qPCR analysis of pluripotency- and DE-associated genes at D1 and D2 of differentiation relative to D0 spheroids on a log10 scale. The results from three independent experiments are presented as the mean ± SD. **E** Graph showing the shift in size of organoids from D0 to D2. The size is expressed as the mean diameter (µm) with error bars showing the standard deviation, representing a total of 570 organoids. Day 0 vs. Day 1 *p* = 0.001, Day 0 vs. Day 2 *p* < 0.0001, Day 1 vs. Day 2 *p* < 0.0001 from Kruskal‒Wallis test with Dunn`s multiple comparisons test. **F** RT‒qPCR analysis of DE and early liver development genes at D7 of differentiation relative to D2 on a log10 scale. The results from three independent experiments are presented as the mean ± SD. **G** Whole-mount immunostaining of D7 organoids showing epithelial (ECAD) and early hepatocyte markers (FOXA2, CK8, HNF4α, AFP) on the outer surface of the organoids. The highlighted area is magnified in Supplementary Fig. 9. **H** Whole-mount immunostaining of D7 organoids showing heterogeneous expression of mesoderm (MESP1)- and mesenchymal (ALCAM, WT1)-associated markers. The highlighted area is magnified in Supplementary Fig. 9. **I** Brightfield image of D20 organoids. Scale bar is 500 µm. **J** Violin plot of D20-D27 organoid diameter from three representative experiments. The solid line represents the mean, while the dotted lines represent the quartiles. **K** RT‒qPCR analysis of both early and later (developmentally) hepatocyte genes at D20 of differentiation relative to D2 on a log10 scale. The results from three independent experiments are presented as the mean ± SD. **L** Whole-mount immunostaining of D20 organoids showing expression of the hepatocyte markers HNF4α and albumin on the outer surface of the organoids. **M** Demonstration of increasing secretion of HGF into the culture medium by the organoids throughout differentiation as measured by ELISAs. The results from three independent experiments are presented as the mean ± SD. All of the above experiments were performed with the hiPSC line AG27. All scale bars are 100 µm unless stated otherwise.

For differentiation of hPSCs into organoids, cells were harvested by incubation with Accutase (Life Technologies) for 10 min at 37 °C until the cells had detached. The cells were pelleted by centrifugation at 300x *g* for 5 minutes at room temperature. After the cells were counted, they were seeded into 125 ml or 500 ml cell culture Erlenmeyer flasks (Corning) at a bulk cell density of 3.5-4 ml of media/million cells, (see^9^) in Essential 8 (Life Technologies) with 10 μM Y-27632 (BOC Sciences). The cells were allowed to self-organize into aggregates for up to 24 hours on an orbital shaker at 70 RPM in a humidified 37 °C, 5% CO_2_ incubator. The conditions for suspension culture (orbital shaker at 70 RPM in a humidified 37 °C, 5% CO_2_ incubator) were utilized for all steps of the differentiation. After aggregate formation, differentiation was initiated to drive pluripotent hPSC aggregates to primitive streak/mesendoderm (Day 1) and further patterned toward definitive endoderm (Day 2) (see Fig. 1A for a schematic overview of the differentiation). For differentiation, the hPSC aggregates were collected from the flask, transferred to a 50 ml conical tube and pelleted by centrifugation for 5 minutes at 300 x g at room temperature. After removal of the supernatant, the aggregates were resuspended in 3.5 ml/million cells of differentiation medium comprised of RPMI 1640 (Life Technologies) with B-27 either with or without insulin (RPMI/B-27 +/-) (Life Technologies) depending on the cell line and 3 or 4 μM CHIR99021 (BOC Sciences). Optimal conditions need to be established for each line based on our previously established protocol^5–8^. The aggregates were then transferred back to the Erlenmeyer flask and incubated for another 24 hours. After 24 hours, the aggregates were collected as described above, and the cell pellet was gently resuspended in the same volume of RPMI/B-27 +/-, without any small molecules, and transferred back to the Erlenmeyer flask. The aggregates were incubated for an additional 24 hours. On Day 2 of differentiation, the cells were directed toward hepatic endoderm (Day 7). The aggregates were collected as described above and resuspended at 3.5 ml/million cells in Knockout DMEM (Life Technologies), 20% (vol/vol) Knockout Serum Replacement (Life Technologies), 1% dimethyl sulfoxide (Sigma-Aldrich), nonessential amino acids (NEAA—Life Technologies), 2-mercaptoethanol (Life Technologies) and Glutamax (Life Technologies) and incubated for 5 days, with a medium change every 48 hours. On Day 7, the resulting organoids were switched to medium for maturation to liver organoids. The medium comprised Lebovitz L-15 base medium with 8.3% fetal bovine serum (FBS-Biowest), 8.3% Tryptose Phosphate Broth (Sigma-Aldrich), hydrocortisone (Sigma-Aldrich), ascorbic acid (Sigma-Aldrich), Glutamax (Life Technologies), 100 nM dexamethasone (Sigma-Aldrich), and 100 nM *N*-hexanoic-Tyr, Ile-6 aminohexanoic amide (Dihexa) (Active Peptide). The organoids were cultured from Day 7 to Day 20, with a medium exchange every 48 hours. Organoids were collected and analyzed or maintained in long-term culture as indicated.

### Fixation of organoids

For transmission electron microscopy (TEM), the organoids were fixed in 1% glutaraldehyde/1% paraformaldehyde (PFA) in 0.12 M phosphate buffer and 0.02 mM CaCl_2_ (pH 7.2–7.5; Sigma) for 4 hours at room temperature, washed in 8% glucose (in 0.12 M phosphate buffer and 0.02 mM CaCl_2_, pH 7.2–7.5) and postfixed in 2% OsO_4_ (in 0.12 M phosphate buffer and 0.02 mM CaCl_2_, pH 7.4; Sigma) for 90 minutes at room temperature. For histology and immunohistochemical detection, the organoids were briefly rinsed in 0.1 M Sörensen buffer (pH 7.4) and immersed in 3% PFA and 0.05% glutaraldehyde in 0.1 M Sörensen buffer (pH 7.4) for 2 hours at room temperature followed by 30 minutes at 4 °C. After a thorough washing in 0.1 M Sörensen buffer (pH 7.4), the organoids were dehydrated and embedded in paraffin. Serial sections (6 µm thick) were cut from paraffin blocks using a microtome, and every tenth slide was stained with hematoxylin-eosin for histological examination.

#### Immunostaining of organoids

Immunohistochemical detection of cytokeratin 18 (CK18) and cytokeratin 19 (CK19) was performed by an indirect two-step method in paraffin-embedded sections. After deparaffinization and rehydration of sections, antigen retrieval was performed in HistoStation (Milestone, Sorisole, Italy). Endogenous peroxidase was blocked in 5% H_2_O_2_ (3 × 10 min), and then, sections were incubated with primary mouse anticytokeratin 18, clone DC10 (DAKO, Glostrup, Denmark; 1:25) or primary mouse anti-cytokeratin 19, clone BA17 (DAKO, Glostrup, Denmark; 1:50) antibody for 1 h at room temperature. After PBS washes, the sections were exposed to antimouse DAKO EnVision+ System-HRP Labeled Polymer (DAKO, Glostrup, Denmark) for 35 min at room temperature. Then, the reaction was developed with 3,3-diaminobenzidine tetrahydrochloride (Sigma-Aldrich). Sections were dehydrated, counterstained with hematoxylin and mounted in DPX (Sigma-Aldrich). Tissue sections were examined with an Olympus BX51 microscope equipped with a DP71 camera.

For whole-mount immunofluorescence and confocal imaging, organoids were fixed in 4% PFA for 60 minutes, pelleted (300x *g* for 5 min, low deceleration) and washed in PBS for 20 min, which was repeated three times. Fixed samples were stored in PBS at 4 °C until needed. Ten microliters of sphere sediment was pipetted onto a coverslip and briefly air-dried, followed by centrifugation at 1000x *g* for 5 min. The fixed organoids were blocked for 1 h in PBS with 0.1% Triton X-100 (Sigma-Aldrich) x 100 (PBS- Triton X-100) and 10% goat serum (Life Technologies). The primary antibody was then diluted at the appropriate dilution in PBS- Triton X-100 containing 1% goat serum and incubated overnight at 4 °C. The primary antibody was removed, and the sample was washed 3 × 20 min in PBS-Triton X-100. All Alexa Fluor secondary antibodies (Life Technologies) were diluted 1:1500 with PBS-Triton X-100, added to the samples for 4 h at 4 °C in the dark and subsequently washed 3 times for 20 min in PBS-Triton X-100. For dual labeling of the samples, the above steps (block, primary, secondary) were repeated. Nuclei were counterstained with DRAQ5 at 1:1500 in PBS for a minimum of 15 min before imaging. A full list of antibodies used are available in Supplementary Table 2.

### Microscopy

Phase-contrast images were obtained using an Axio Primovert upright light microscope (Zeiss). Images were captured using Zen Software (Zeiss). All scale bars represent 100 μm unless otherwise stated in the figure legends. Confocal images were obtained using Olympus FV1000 and Andor Dragonfly microscopes, and images were captured using Olympus Fluoview and Fusion, respectively, and compiled using ImageJ software.

#### Spinning disk confocal microscopy

For clear organoid visualization, we used the ultrafast confocal system IXplore SpinSR Olympus (Olympus, Tokyo, Japan). We utilized our previously published imaging settings^10^. The imaging system consists of the following units: an inverted microscope (IX83; Olympus, Tokyo, Japan) and a spinning disc confocal unit (CSUW1-T2S SD; Yokogawa, Musashino, Japan). Fluorescence data for image reconstruction were collected via either a 100 x silicone immersion objective (UPLSAPO100XS NA 1.35 WD 0.2 silicone lens, Olympus, Tokyo, Japan) or a 20 x objective (LUCPLFLN20XPH NA 0.45 air lens, Olympus, Tokyo, Japan). The following lasers were used to excite fluorophores: 405 nm laser diode (50 mW) and 488 nm laser diode (100 mW). Confocal images were acquired at a 2048 × 2048-pixel resolution. Optical sections were acquired at 1.5 μm and 250 nm intervals along the z-axis for 3D reconstruction of 20x and 100x objectives, respectively. The fluorescent images were collected by appropriate emission filters (BA420-460; BA510-550; Olympus, Tokyo, Japan) and captured concurrently by two digital CMOS ORCA-Flash4.0 V3 cameras (Hamamatsu, Hamamatsu City, Japan). Fluorescence confocal images were acquired using cellSens software (Olympus, Tokyo, Japan). Icy open source software was used for image processing and 3D reconstruction^11^.

### Cryosectioning

PFA-fixed organoids were transferred to a cryomold, embedded in OCT compound (Thermo Fisher Scientific) and cooled to −80 °C in a bath of isopropanol on dry ice. The cryo-embedded organoids were then sectioned at 50 μm on a cryotome and transferred to slides. Slides were stored at −80 °C until immunostaining and imaging as described above.

#### Transmission electron microscopy

The fixed organoids (described above) were rinsed and incubated overnight in 10% sucrose (in water) at 4 °C, and the organoids were dehydrated in graded alcohols (50%, 75%, 96%, 100%), cleared in propylene oxide and embedded in a mixture of Epon 812 and Durcupan (Sigma; polymerization for 3 days at 60 °C). First, semithin sections were cut on an Ultrotome Nova (LKB, Sweden) and stained with toluidine blue. Subsequently, ultrathin sections were cut on the same ultramicrotome, collected onto formvar carbon-coated copper grids, counterstained with uranyl acetate and lead citrate and examined under a JEOL JEM-1400Plus transmission electron microscope (at 120 kV, JEOL, Japan).

#### LSEC functionality testing

Day 21 organoids were transferred to a 6-well suspension plate at 3 ml/well. In separate wells, either Alexa Fluor™ 488 AcLDL (Thermo Fisher, L23380) or FITC-FSA (Gift from Karen Sørensen), both at 2 µg/ml, was added to the culture medium. The samples were incubated at 37 °C for both 15 and 75 minutes before being washed twice in culture medium and fixed as described previously. Organoids were then immunostained with endothelial markers as described above.

### RNA

Two methods were employed to isolate RNA. (i) Cells were collected for RNA isolation from 2D controls by washing the cells once with DPBS^−/−^, followed by scraping the cells into DPBS^−/−^. The resulting cell suspension was pelleted by centrifugation at 300x *g* for 1 minute at room temperature. The supernatant was carefully removed, and TRIzol (Life Technologies) was added to lyse the cells. For organoids, RNA isolation was performed by removing 2 ml of suspension culture medium from the Erlenmeyer flasks and collecting the organoids by centrifugation at 300x *g* for 5 min at room temperature. The supernatant was carefully removed, and organoids were washed with 5 ml of DPBS^−/−^ and repelleted. DPBS^−/−^ was gently removed, and TRIzol was added to lyse the organoids. TRIzol samples were then either processed immediately for RNA isolation according to the manufacturer’s instructions or stored at −80 °C for subsequent processing. RNA was quantified using a NanoDrop ND-1000 Spectrophotometer (NanoDrop). For vitamin K-dependent enzyme analysis, total RNA was isolated using the MagMAX™-96 Total RNA Isolation Kit on a MagMAX™ Express-96 Deep Well Magnetic Particle Processor as described by the manufacturer (both from Thermo Fisher Scientific, Waltham, MA, USA).

### cDNA synthesis

Five hundred nanograms of RNA was used as a template for reverse transcription to cDNA. cDNA synthesis was performed using the High-Capacity Reverse Transcriptase Kit (Life Technologies) with random primers, following the manufacturer’s instructions for reactions without RNase inhibitor.

### Gene expression analysis with RT‒qPCR

Gene expression was analyzed via reverse transcriptase quantitative polymerase chain reaction (RT‒qPCR) using TaqMan probes (Life Technologies) or SSO Universal Probes Master Mix (Bio-Rad). For a complete list of probes used in this study, please see Supplementary Table 2. All samples were analyzed in triplicate. Data are presented as the average of three independent experiments +/- the standard deviation.

### CYP450 activity and induction

Analysis of cytochrome P450 (CYP) basal activity and inducibility was performed as previously described with several modifications for organoid cultures^6^. Briefly, 2D HLCs and organoids were induced from Day 20 onward of differentiation with prototypical CYP450 inducers: for CYP3A4, we cultured cells with 25 μM rifampicin and 100 μM omeprazole for CYP1A2 (Sigma-Aldrich). Prior to starting the inductions, the cells/organoids were washed with DPBS^−/−^ 4 times to remove hydrocortisone and dexamethasone. After washing, the inducers were added to L-15 medium as described above, minus hydrocortisone and dexamethasone. The induction medium was refreshed every 24 h for 3 days. Seventy-two hours postinduction, the cells were assayed for CYP1A2 and CYP3A4 activity using the P450-Glo CYP3A4 (Luciferin-PFBE) Cell-based/Biochemical Assay kit (Promega, Cat. no. V8902) and the P450-Glo CYP1A2 Induction/Inhibition Assay kit (Promega, Cat. no. V8422) according to the manufacturer’s instructions. Data were normalized to 1 million hepatocytes and are presented as the average of three independent experiments +/- the standard deviation.

### Heroin metabolism

After 21 days of differentiation, 50 organoids per well were loaded in triplicate into a 96-well plate and treated with culture media with 10 µM heroin for 1, 3, 6, and 24 hours. For controls, we used culture media without organoids; these samples were assessed in parallel to measure heroin degradation throughout the experiment. To stop metabolism at each time point, we transferred the samples to a new 96-well plate prefilled with formic acid (final conc. 0.1 M) along with internal standards. The samples were centrifuged for 10 min at 1000x *g* at 4 °C. The supernatants were transferred to autosampler vials and analyzed for heroin, morphine, and morphine-3β-D-glucuronide (M3G) using an Acquity UPLC system (Waters, Milford, MA) coupled to a Xevo-TQS triple quadrupole mass spectrometer with an electrospray ionization interface (Waters) based on a method previously described^12^. Data acquisition, peak integration, and quantification of samples were performed using MassLynx 4.0 SCN509 software (Waters Corp., Milford, MA, USA).

### Coagulation factor studies

For coagulation studies, hepatic organoids were cultured in L15 medium with Vit K (Konakion Roche) 5 µg/ml for at least 48 h before harvesting.

### FVII activity

Factor VII (FVII) activity in the cell medium was determined using the Human FVII Chromogenic Activity Kit (Nordic BioSite AB, Täby, Sweden) according to the manufacturer’s instructions. Primary hepatocytes (Thermo Fisher Scientific) were used as controls. The functional activity of FVII from supernatants of iPSC-HO was assessed by two methods: a) western blotting after activation with tissue factor (TF, STA Neoplastin) and CaCl_2_ and incubation with antithrombin (AT), purified from human plasma, (Grifols®, Spain) and unfractionated heparin (Hospira®) to measure the ability of FVII to become active and form a complex with AT; and b) thrombin generation (CAT, Stago®, Valencia, Spain) of supernatants derived from organoids with and without FVII-depleted plasma. Briefly, 40 µl of organoid supernatants was added to 40 µl of FVII-depleted plasma and activated with 5 pM TF. Fluorescence measurements started just after the addition of Fluka® reagent diluted in a commercial buffer containing calcium. As controls, we used only 80 µl of FVII-depleted plasma and 40 µl of serum-free medium (SFM L15) with 40 µl of FVII-depleted plasma, with activation by 5 pM TF. Fluorescence measurements were recorded for 60 min in a fluorimeter (Stago ®, Valencia, Spain).

### Western blot analysis of FII, AT, A1AT and FVII

Intracellular levels of FII were determined by western blots (WBs). Briefly, hepatic organoids were lysed in T-PER^TM^ buffer (Thermo Fisher Scientific), and lysates were collected by centrifugation at 8000x *g* for 10 min. Equal amounts of proteins from lysates were separated by SDS‒PAGE using Mini-PROTEAN® TGX™ 10% Precast Gels (Bio-Rad, Hercules, CA, USA) before transfer onto a Sequi-Blot PVDF membrane (Bio-Rad) using the Mini Trans-Blot Electrophoretic Transfer Cell system (Bio-Rad). The membranes were incubated overnight at 4 °C with the primary antibody anti-FII (Novus Biologicals, Centennial, CO, USA) or beta-actin (Sigma-Aldrich, Saint Louis, MO, USA). The membranes were washed and then incubated with the appropriate horseradish peroxidase (HRP)-conjugated secondary antibody (Santa Cruz Biotechnology, Dallas, TX, USA) for 1 h at room temperature. The blots were developed using Radiance Plus chemiluminescent substrate (Azure Biosystems, Dublin, CA, USA), and the signals were quantified using the ImageQuant LAS-4000 mini-Imager (GE Healthcare, Chicago, IL, USA). Primary hepatocytes were used as controls.

AT, alpha-1-antitrypsin (A1AT) and FVII were evaluated by western blots on 8% polyacrylamide gel electrophoresis performed under 10% SDS denaturing conditions in the presence 0.05 M reducing agent. AT, A1AT and FVII were immunostained with rabbit anti-human AT (A9522, Sigma-Aldrich), anti-human A1AT (Dako Diagnostics, Denmark) and anti-human FVII (AF2338, R&D) polyclonal antibodies, respectively.

### Evaluation of N-glycosylation of secreted proteins

N-glycosylation was evaluated by comparative WBs under basal conditions and after digestion with neuraminidase (α(2 → 3, 6, 8, 9)) from *Arthrobacter ureafaciens*, Sigma-Aldrich), following the manufacturer’s instructions, and with N-glycosylase F (PNGase F, Sigma-Aldrich, Madrid, Spain). Cell supernatants (up to 200 µg of glycoprotein in a volume of 35 µl) were denatured with 10 µl of 250 mM phosphate buffer and 2.5 µl of 2% SDS with 1 M 2-mercaptoethanol, heated at 100 °C for 5 minutes and cooled. Triton X-100 (2.5 µl of 15%) (v/v) was added. Then, 2.0 µl of PNGase F (≥5000 units/ml) was added and incubated overnight at 37 °C. Samples were run in SDS‒PAGE and detected as described above.

### Serum protein analysis via ELISA

ELISAs for human albumin (Bethyl Laboratories, Inc.), human A1AT (Abcam cat# ab108799) and hepatocyte growth factor (Antibodies-online) were used as described by the supplier. Colorimetric readings were taken at the specified wavelength on a SPECTRAmax PLUS 384. Values were derived from standards using appropriate lines of best fit generated on Softmax pro software. For clotting factors, organoids were collected by centrifugation for 10 minutes at 300x *g*, and the cell medium was collected. Hepatic organoids were lysed in T-PER^TM^ buffer (Thermo Fisher) containing Halt protease and phosphatase inhibitor cocktail 1X (Thermo Fisher). FVII and FX antigens (FVIIAg and FXAg) were measured in the cell medium using an FVII ELISA kit and FX ELISA kit (Abcam), respectively. FII, AT, Protein C, and Protein S were measured using Procarta-plex coagulation 6 plex panel 1 and coagulation 4 plex panel 3 (Thermo Fisher Scientific). Primary hepatocytes were used as controls. The FVIIAg and FXAg levels (ng/ml) were normalized to 1 × 10^6^ cells, and the ratio of organoid/primary hepatocytes was calculated.

### Oleic acid accumulation assay

Oleic acid (1 M, Sigma-Aldrich) was diluted with NaOH (Sigma-Aldrich) and heated at 70 °C for 30 minutes to form a 20 mM sodium oleate solution. This solution was then diluted with a 5% BSA/PBS solution at 37 °C to form a 5 mM sodium oleate/BSA complex. This solution was further diluted to 300 μM in culture media and incubated with organoids for 5 days, changing the media each day. After fatty acid treatment, the organoids were incubated with 3.8 μM BODIPY 493/503 (Life Technologies) for 30 min in culture medium at 37 °C and then washed two times with PBS before being replaced with fresh culture medium containing DRAQ5 (Thermo Fisher) at 1:1500. Organoids were then imaged on a confocal microscope as described above.

### Sample preparation, processing and data processing of proteomics data

#### Patient liver samples

Five patient samples were collected from liver explants from patients undergoing LTX at Oslo University Hospital. The samples were stored in liquid nitrogen. The regional ethics committee approved the use of the patient material (REK 2012-286) in accordance with the Declaration of Helsinki. All participants provided written informed consent.

Samples were processed as follows: the proteins were precipitated with acetone/TCA (Sigma-Aldrich). The pellets were resuspended in 8 M urea in 50 mM NH_4_HCO_3_, and the proteins were reduced, alkylated and digested into peptides with trypsin (Promega). The resulting peptides were desalted and concentrated before mass spectrometry by the STAGE-TIP method using a C18 resin disk (3 M Empore). Each peptide mixture was analyzed by a nEASY-LC coupled to QExactive Plus (ThermoElectron, Bremen, Germany) with an EASY Spray PepMap®RSLC column (C18, 2 µl, 100 Å, 75 µm x 25 cm) using a 120-minute LC separation gradient.

The resulting MS raw files were submitted to MaxQuant software version 1.6.1.0 for protein identification. Carbamidomethyl (C) was set as a fixed modification, and acetyl (protein N-term), carbamyl (N-term) and oxidation (M) were set as variable modifications. A first search peptide tolerance of 20 ppm and a main search error of 4.5 ppm were used. Trypsin without the proline restriction enzyme option was used, with two allowed miscleavages. The minimal unique+razor peptide number was set to 1, and the allowed FDR was 0.01 (1%) for peptide and protein identification. The UniProt database with ‘human’ entries (October 2017) was used for the database searches.

Proteins with log2(intensity) > 10 average intensity value were defined as “expressed proteins”. The Pearson correlation coefficient between the liver and organoid was calculated from log2(intensity) with the cor.test function in R. Differential expression of proteins between the liver and organoid was defined as a more than 2-fold change and *p* < 0.05 by a two-sided T test. Gene Ontology analysis was conducted with the GOstats Bioconductor package^13^. Multiple test correction was performed by the Benjamin-Hochberg method with the p.adjust function in R.

### Library preparation and scRNAseq data processing

scRNA-seq libraries were prepared from the liver organoids at Day 48 with Chromium Single Cell 3’ Reagent Kits (version 2-10x Genomics) as described previously^14^. Conversion to fastq format, mapping/UMI counting in human genome (hg19) and data aggregation were implemented by *mkfastq*, *count* and *aggr* functions with default parameters in CellRanger software (v2.1.0). Subsequent data processing, such as batch effect normalization, was performed by Seurat software (v3.1.0)^15^. In each replicate, the feature UMI count was normalized to the total count and multiplied by 10,000. The top 2,000 highly variable features (HVFs) were then identified by variance stabilizing transformation. Anchor cells across different scRNAseq libraries were identified with HVFs under 20-dimensional spaces from canonical correlation analysis and used for the transformation of multiple scRNAseq datasets into a shared space. Gene expression values were scaled for each gene across all integrated cells and used for principal component analysis (PCA). Twenty PCs were further assigned into two-dimensional space using uniform manifold approximation and projection (UMAP) and used to identify cell clusters. Differentially expressed genes in each cluster were identified with a more than 1.25-fold change and *p* < 0.05 by two-sided T test. Overrepresented GO terms were identified by GOstats (v2.24.0)^13^. Multiple test correction was performed by the Benjamin-Hochberg method with the p.adjust function in R.

The cluster labels were assigned by cell type-specific markers and GO terms (Supplementary Fig. 2c, e). Nine out of 22 clusters were first separated by the overrepresentation of “extracellular matrix (GO:0031012)”, which is a feature of stellate and endothelial cells. Active (ASTs) and resting stellate cells (RSTs) were defined by genes involved in “mitotic nuclear division (GO:0140014)” and its markers (MGP and ELN). Non stellate clusters were labeled endothelial cells (ECs) and further divided into liver sinusoidal endothelial cells (LSECs) and macrovascular endothelial cells (MVECs) by the absence and presence of vasculogenesis markers (KDR and HAND1)^16^. Seven of 13 other clusters were assigned hepatocyte (HEP), cholangiocyte (CHO), Kupffer cell (KPC) and Kupffer precursors (KPP) using the enrichment of GO terms “Cholesterol homeostasis (GO:0042632)”, “Keratinization (GO:0031424)”, “Phagocytosis (GO:0006909)” and “Hematopoietic stem cell differentiation (GO:0060218)”, respectively. Five clusters were assigned as peripheral nervous system with the expression of neuronal lineage markers (SOX2 and PAX6) and further divided into neuron (Neu), glia (Glia), neuro progenitor (NPC) and cilia-bearing cell (CBC) with “axon development (GO:0061564)”, “glial cell development (GO:0010001)”, “mitotic nuclear division (GO:0140014)” and “cilium assembly (GO:0060271)”, respectively^14^. We could not identify any unique marker or relevant GO terms in one cluster and labeled it unknown (UN). The cluster labeling strategy is schematically represented in Supplementary Fig. 2b.

Public transcriptome profiles were downloaded from the NCBI Gene Expression Omnibus database. The single-cell transcriptome of the liver organoid from Ouchi *et al*. (GSE130073)^3^ and the human liver atlas (GSE124395)^17^ were merged with our scRNAseq data and plotted into the shared UMAP space by Seurat as described above. Clusters, which are mainly composed of CD45^+^ cells and unique to the human liver atlas, were labeled as “other immune cells”. Genes were sorted by the difference in the average expression of all cells between our and Ouchi et al. liver organoids and used for GSEA (v2.2.2) of REACTOME genes without collapsing the gene set^18^. Cell-type-specific gene signatures were constructed from bulk RNA-seq in primary hepatocytes (GSE98710, GSE112330 and GSE135619)^19,20^, biliary tree stem cells (GSE73114)^21^, stellate cells (GSE119606)^22^ and endothelial cells (GSE114607)^23^. The RNA-seq reads were aligned to the hg19 human genome by TopHat (v2.2.1) with default parameters^24^. The mapped reads were counted in each gene by HTSeq software (v0.9.0) with options “-s no -f bam”^25^.

The factors of technical variations across multiple transcriptome datasets were minimized by the RUVs function in RUVSeq (v1.8.0)^26^. Subsequently, differentially expressed genes in each cell type were identified by DESeq2 (v1.14.1). For evaluation of the enrichment of the cell-type specific genes, genes were sorted in individual cells by relative expression level to average of all cells and used for GSEAPY software (v0.9.3) with options “--max-size 50000 --min-size 0 -n 1000”. Hepatic zone-specific genes were obtained from transcriptome profiles of hepatocytes from laser-microdissected human livers (GSE105127)^27^. After processing the bulk RNA-seq, the zone-specific genes were defined with a more than 1.5-fold change and *p* < 0.05 by a two-sided T test. The enrichment was evaluated by GSEAPY software with preranked genes in individual cells relative to all cells in all hepatocyte clusters.

For investigation of the transcriptional bias between LSECs and MVEC, cells from EC clusters were ordered in pseudotemporal spaces by Monocle (v2.99.3). Briefly, the Monocle object was first constructed from the UMI count matrix for cells in EC clusters and preprocessed according to the instructions. We then replaced data in “normalized_data_projection” and “reducedDimW” with nontransposed and transposed PCA dimensional matrices. In addition, the “reducedDimS”, “reducedDimA” and “reducedDimK” slots were replaced with the transposed UMAP dimensional matrix. The principal graph was learned by the learnGraph function with “RGE_method = 'DDRTree', close_loop=T, prune_graph=F, euclidean_distance_ratio=5”. Subsequently, cells were ordered according to the trajectory by the orderCells function using MVEC1 as a root cluster. Differentially expressed genes were identified by the differential GeneTest function with the model “~sm.ns(Pseudotime)”. Finally, genes with q < 1e-50 were selected as EC ordering-dependent genes and used for GO analysis by GOstats as described above.

### Animal work

Male and female NOD. Cg-Prkdc^scid^ Il2rg^tm1Wjl^/SzJ (NOD *scid* gamma, NSG) mice (purchased from The Jackson Laboratory, Bar Harbor, ME, USA) were housed in a Minimal Disease Unit at the animal facility at Oslo University Hospital Rikshospitalet, Oslo, Norway, with a 12-hour light–dark cycle and *ad libitum* access to water and standard rodent diet. All experiments were performed with co-housed age-matched mice. Mice undergoing surgery were not fasted and were 15 weeks of age at the time of surgery. All animals received human care, and the animal experiments were approved by the Norwegian National Animal Research Authority (project license no FOTS 19470) and performed according to the European Directive 2010/63/EU, the Animal Research: Reporting of In Vivo Experiments guidelines and The Guide for the Care and Use of Laboratory Animals, 8th edition (NRC 2011, National Academic Press).

### Implantation of human liver organoids under the rodent kidney capsule

Male and female immunodeficient NSG mice were used in this study. The transplantation of the organoids under the kidney capsule or the sham laparotomy was performed as described in^28^ with some modifications. In brief, the procedure was performed using proper multimodal analgesia with s.c. administration of local analgesia (Marcain, 0.07 ml/10 g BW) in combination with s.c. administration of buprenorfin (0.1 mg/kg) before surgery and general anesthesia with i.p. injection with FD2 (fentanyl/domitor/dormicum) and Antisedan (antagonist) post-surgery. Following a sterile preparation of the left flank, a 1.5 cm incision was made midway between the last rib and the iliac crest and approximately 0.5 cm parallel and ventral to the spine^28,29^. The left kidney was slowly externalized through the abdominal incision using sterile cotton swabs, immobilized using nontraumatic forceps and moisturized with warm sterile saline. The injection site was located at the upper lateral side of the kidney, and a 1 ml syringe with a 25 G needle containing either the organoid suspension or pure Matrigel (Thermo Fisher Scientific) (sham surgery) was gently pushed under the capsule toward the inferior pole of the kidney to avoid perforation and damage to the blood vessels. Fifty to eighty microliters of organoid suspension or Matrigel matrix was very slowly discharged under the kidney capsule, and the needle was simultaneously slowly pulled out of the capsule to avoid backflow. Next, the kidney was returned to the body cavity, the abdominal wall was closed with sutures, and the skin incision was closed with 7 mm wound clips. After surgery, the mice were examined daily the first week for normal wound healing, with weight measurements and general well-being, thereafter once a week. Once every week, blood was sampled from the saphena vein for serum marker measurement.

### Statistical analysis

Data are reported as the mean and standard deviation (if normally distributed) or as the median and interquartile range. Comparisons of two groups were performed using unpaired Student’s t test or Mann‒Whitney U test. For comparisons with more than two groups, Kruskal‒Wallis with Dunn`s multiple comparisons test was applied. Statistical analyses were performed with Graph Pad Prism 9.4.1 for Windows (GraphPad Software, Inc., San Diego, California).

## Results

### Differentiation of human pluripotent stem cells into liver organoids

We employed a cocktail of small molecule mimetics in a developmentally relevant sequence to mimic in vivo liver development (Fig. 1A)^5,6,30^. Upon initiating organoid formation, we observed rapid aggregation of the hPSCs post-single-cell seeding, generating aggregates with an average diameter of 116 ± 41 μm, which expressed the pluripotency markers OCT4, SOX2 and NANOG over a 24-hour period (Fig. 1B and Supplementary Fig. 9). For initiation of the developmental program, aggregates were challenged with a pulse of WNT signaling via CHIR99021 (CHIR), leading to an exit from pluripotency and a transition through primitive streak, resulting in aggregates of 128 ± 57 μm (24 hours post-CHIR treatment). The WNT signal was removed after 24 hours, and by Day 2, the aggregates exhibited markers of both mesoderm and definitive endoderm (DE): *T*, *GSC*, *FOXA2*, *SOX17*, *HHEX* and *CER1* (Fig. 1C and D). During the initial 2 days of differentiation, we observed a shift in the size of the organoids, resulting in mesendodermal aggregates with an average size of 159 ± 46 μm at Day 2 (Fig. 1E). The mesendodermal aggregates were then subjected to hepatic specification until Day 7, and the resulting organoids were characterized for the presence of hepatic markers. We observed increased expression of *AFP*, *CEBPa*, *HNF4α* and *TTR* coinciding with decreased expression of DE markers such as *HHEX*, *SOX17* and *GATA4* (Fig. 1F). Expression of the T-box family protein *TBX3* was observed (Fig. 1F); this molecule is a factor involved in hepatic endoderm delamination and invasion into the adjacent *septum transversum* mesenchyme (STM), resulting in the mixing of these two germ layers and the formation of the liver bud. Using immunostaining, we revealed further characteristics of a mixed lineage liver bud stage. The outer epithelial layer of cells expressed ECAD (CDH1), FOXA2, CK8, HNF4α and AFP, indicative of an early hepatic phenotype (Fig. 1G, Supplementary Fig. 1 and Supplementary Fig. 9). These epithelial markers were localized to the outer surface and not the core of the organoids (Supplementary Fig. 1).

We characterized the nonepithelial cores of Day 7 organoids, where we observed a population of MESP1-positive cells, indicative of mesodermal STM^31^ (Fig. 1H and Supplementary Fig. 9). During development, a mesodermal MESP1^+^ population gives rise to mesothelial/submesothelial populations marked by ALCAM and Wilms Tumor (WT1), which in turn give rise to hepatic stellate cells (HSCs)^31,32^. We also identified ALCAM- and WT1-positive populations within the mesenchyme of the organoids, marking the putative mesothelial/submesothelial populations (Fig. 1H and Supplementary Fig. 9). The early hepatic organoids were directed to a mature liver-like stage over a 14-day period by the addition of differentiation media with the small molecule Dihexa, a hepatocyte growth factor (HGF) small molecule mimetic^4,5,29^, resulting in the formation of organotypic 3D structures (Fig. 1I) with an average size of 250 μm (Fig. 1J). The liver organoids exhibited markers of hepatic maturation and tissue-like cellular complexity. Analysis by RT‒qPCR demonstrated the expression of hepatic markers such as *ALB*, *A1AT*, and *ASGR1* as well as genes enriched in the liver such as *APOA2*, *TDO2, HNF4α* and *TTR* and those involved in xenobiotic metabolism cytochrome P450 (*CYP) 3A4* and *CYP3A7* (Fig. 1K). Immunofluorescence staining demonstrated that the hepatocytes were located on the surface of the organoids visualized with HNF4α and ALB staining (Fig. 1l). Organogenesis is initiated when the epithelium interacts with an early mesenchymal population during liver development^33^. This process is driven by a number of paracrine factors, including the essential morphogen HGF^34^. We assessed the production of HGF during differentiation and noted that the secretion of HGF increased throughout organoid differentiation (Fig. 1M). This process gives further support to the developmental accuracy of organoid differentiation, as HGF is secreted early in development by the mesenchymal population and later by their derivatives, e.g., hepatic stellate cells (HSCs). This process is shown in vivo to drive the expansion and maturation of the liver bud, suggesting that it potentially fulfils a similar role in our organoids^35^.

### Single-cell transcriptome analysis of iPSC-derived liver organoids

**Fig. 2:**
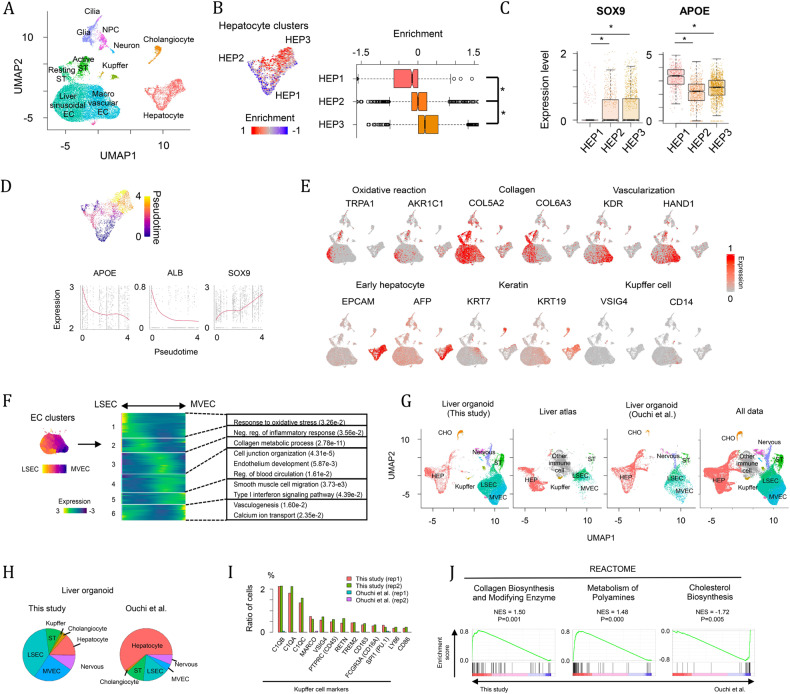
Single-cell RNA sequence analysis of liver organoids. **A** UMAP plot of single cells distinguished by cell type. **B** GSEA of gene signatures of periportal hepatocytes in hepatocyte clusters. The enrichment was compared across three hepatocyte clusters. * *p* < 0.05 by two-sided Student’s t test. **C** Differential expression of SOX9 and APOE across hepatocyte clusters, * *p* < 0.05 with two-sided Student’s t test. **D** Pseudotemporal ordering of hepatocyte clusters. SOX9 and APOE expression levels are positively and negatively correlated with pseudotime. **E** Heatmap showing the expression of genes related to oxidative stress, collagen, vascularization, early hepatocyte development, keratin and Kupffer cell development. **F** Endothelial cell ordering from LSECs to MVECs. The heatmap represents the expression pattern of genes, which are dependent on the EC ordering and categorized into six groups. Significant GO terms in each gene group are also shown. **G** UMAP plot of single cells derived from our and the Ouchi et al. liver organoids^3^ and human liver^17^. **H** Pie charts representing cell composition in our and the Ouchi *et al*. liver organoids. **I** Ratio of cells expressing Kupffer cell markers. **J** GSEA of pathway-related genes between our and Ouchi *et al*. liver organoids^3^. All of the above experiments were performed with the hiPSC line AG27.

To further dissect the cellular diversity within the liver organoids, we profiled the single-cell transcriptomes of a total of 21,412 cells by scRNAseq. A total of 22 clusters were detected and systematically assigned into liver cell types by unique markers, Gene Ontology (GO) functions and reference transcriptome profiles (Fig. 2A and Supplementary Fig. 2a-e)^19–23^. We identified three hepatocyte clusters (HEP1-3) with substantial expression of glycerolipid or cholesterol metabolic genes^36^. In the mammalian liver, hepatocytes are hexagonally arranged into hepatic lobules that display a gradient microenvironment of oxygen, nutrients, hormones and secreted proteins from pericentral to periportal zones^37^. To evaluate the spatial heterogeneity of the hepatocytes within the liver organoid, we performed gene set enrichment analysis (GSEA) of gene signatures for human periportal hepatocytes to individual cells in the hepatocyte cluster (Fig. 2B)^27^. The enrichment of periportal gene signatures was significantly different across the three hepatocyte clusters. In particular, the HEP3 cluster was closest to the periportal zone, while HEP1 was more distant, suggesting that gene expression zonation of hepatocytes was present in the liver organoids. Next, we plotted the normalized enrichment scores of periportal genes and showed a significant difference across the three hepatocyte clusters (Fig. 2C and D). However, at the individual gene level with respect to well-defined zonal genes, these showed low expression, and only a few genes were differentially expressed across the hepatocyte cluster. Therefore, we demonstrate that partial zonation is present *via* markers differentially expressed across hepatocyte clusters. For example, several zonation markers, such as CYP2E1 and LGR5, were expressed at low levels in liver organoids. We speculate that this is because our organoids mimic the developing liver and are not yet fully mature, i.e., at an early postnatal stage, where full zonation of the acinus architecture is not established at birth and takes many years to fully organize^38^.

The endothelial cell (EC)-like clusters were characterized by ECM genes and reference transcriptome annotation (Fig. 2E and F) and divided into liver sinusoidal endothelial cells (LSECs) and macrovascular endothelial cells (MVECs) by the expression pattern of vascularization markers (Fig. 2F). To infer continuous EC heterogeneity, we ordered cells from EC clusters along with their gene expression patterns (Fig. 2F). Six co-expression modules were transiently expressed with EC ordering and were involved in different cellular events. The modules biased to LSECs included genes related to oxidative reactions and anti-inflammatory responses, which are characteristics of the liver sinusoid^39,40^. In contrast, genes involved in smooth muscle and calcium transport were significantly enriched in MVEC-biased gene modules. We next explored LSEC zonation, mapped gene signatures of LSECs in central and periportal zones^41^ and observed no significant differential expression of LSEC markers and gene signatures in the EC clusters, indicating that LSEC zonation has not yet been established in our organoid model. This finding is not unexpected, as a study by Liang et al.^42^ only observed pericentral and periportal LSEC zonal populations after Day 56 in rodents. In neonates, these researchers observed heterogeneity in the premature liver, without clear zonation separation. This finding indicates that zonation profiles are being progressively built up during postnatal liver development.

Interestingly, in addition to the above liver cell types, we also identified clusters expressing genes for the development of the peripheral nervous system (*SOX2*, *PAX6*, *STMN2*) (Fig. 2A, G and H and Supplementary Fig. 2b and e). This result is not surprising, as the human liver is highly innervated^43^, and neural crest cells were shown to appear in the epiblast prior to emergence of definitive ectoderm and mesoderm^44,45^.

We next examined the consistencies and differences with the primary liver and against another liver organoid protocol described by Ouchi et al. ^3^. This protocol was one of the first for iPSC-derived liver organoids, allowing the comparison of the cell composition and transcriptional profiles. The scRNAseq data were compared against scRNAseq of FACS-sorted human adult liver cell populations, liver organoids derived from the Ouchi protocol and data from all sources in the shared UMAP space (Fig. 2G and Supplementary Fig. 2f)^3,17^. The liver cell types (hepatocytes, HSCs, Kupffer cells and endothelial cells) in our liver organoid are close to cells derived from the human liver, indicating similar gene expression profiles of these cells between our organoid and primary human liver. The liver organoids from the two different protocols exhibited similar cellular compositions but displayed differences in the amount of nonendodermal cell types (Fig. 2H). For example, our protocol produces a high number of endothelial cells, while more than half of cells are committed to hepatocytes in the Ouchi *et al*. organoids. In addition, peripheral nervous system-like cells were detectable in both studies. Our comparative analysis revealed that the liver organoids displayed the generation and maturation of Kupffer cells (Fig. 2I), which were not detectable in the Ouchi study. Cells expressing cell surface markers (*MARCO*, *CD45*, *CD163*, *CD16A*, *LY86* and *CD86*), complement components (*C1QA*, *C1QB* and *C1QC*) and macrophage regulators (*VSIG4 TREM2* and *PU.1*) were enriched in our protocol. For further comparison, we analyzed preferential pathways between the two protocols. GSEA for the REACTOME database revealed that collagen and polyamine metabolic genes were significantly enriched in our protocol (Fig. 2J). In contrast, cholesterol biosynthetic genes were enriched in Ouchi and colleagues’ protocol, potentially due to the higher number of hepatocytes in this protocol.

### Proteomic analysis of iPSC-derived liver organoids

**Fig. 3:**
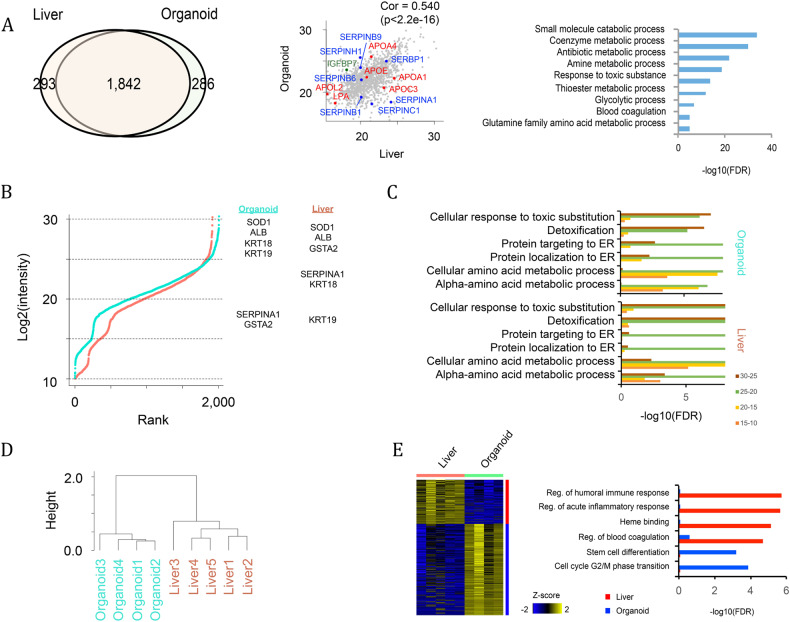
Proteomic analysis of liver organoids. **A** Venn diagram representing proteins shared between in vivo liver and in vitro organoids. The correlation is also shown with the scatterplot, and the overrepresented GO terms of the shared proteins are shown by a bar graph. **B** Ranked plot of log2(intensity) of proteomics data in organoid (blue) and liver (red). Proteins are categorized by four intensity ranges (25~, 20–25, 15–20 and 10–15). Representative liver markers in each intensity range are shown in the right panel. **C** Bar plot showing overrepresented Gene Ontology terms in each intensity range. **D** Unbiased clustering of proteomic data. Clustering was performed to log2(intensity) value of all expressed genes (log2(intensity)>10). **E** Differentially expressed proteins and their overrepresented GO terms. All of the above experiments were performed with the hiPSC line AG27.

Above, we explored the composition of the organoids at the transcriptome level. Next, we performed global proteomic analysis. This analysis revealed that 1,842 out of 2,461 detected proteins were expressed in both human primary liver (biopsies from human liver) and in vitro generated liver organoids. GO analysis indicated that these enriched proteins were involved in a variety of metabolic pathways and functions, including blood coagulation (F2, SERPINC1, SERPING1, FGG, FGA, etc.) and glutamine family amino acid metabolic processes (ARG1, ASS1, FAH, GLUL, GFPT1, GOT1, GOT2, etc.) (Fig. 3A and Supplementary Table 1). We also used iBAQ plots to illustrate key liver markers in each intensity range (Fig. 3B). Liver markers such as SOD1 and ALB showed the highest expression in both organoids and the liver. Other liver markers, such as KRT18 (CK18), GSTA2 and SERPINA1, were also expressed in both the organoid and liver, but their intensity varied between the organoid and liver. GO analysis indicated that similar protein sets were enriched in organoid and liver samples. For example, proteins related to “response to toxic substitution” and “detoxification” were enriched in the highest expression range (~25) (Fig. 3C). Overall, these results support that key liver proteins are commonly expressed in both organoid and liver samples. Next, we used unbiased clustering of the proteomic data, and clustering was performed on the log2(intensity) value of all expressed genes (log2(intensity) > 10) (Fig. 3D). We noted that a separation of the organoid and liver was robustly observed by various cutoff values (e.g., 10, 15, 20) of the expressed genes. A heatmap shows differentially expressed proteins between organoids and liver, identifying 200-400 proteins that were differentially expressed (Fig. 3E). These differences were proteins related to blood cells (e.g., immune response and heme-binding), which were significantly enriched in primary liver (Fig. 3E). In the organoids, cell cycle and early developmental genes were enriched.

### iPSC-derived liver organoids contain hepatic parenchymal cells

**Fig. 4:**
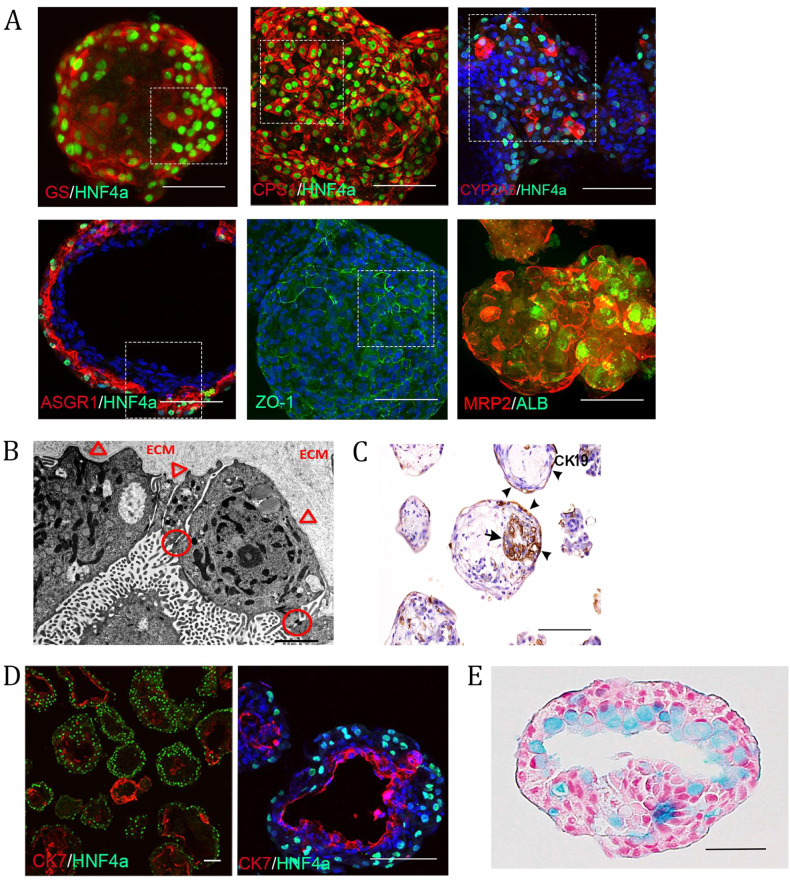
Liver organoids contain the parenchymal cell types of the liver. **A** Immunostaining showing the expression of various maturity- and polarity-associated hepatocyte markers in the outer most layer of the organoids (whole mount except for ASGR1/HNF4α which are from 50 μm thick cryosections). Scale bars are 100 μm, and the highlighted area is magnified in Supplementary Fig. 9. **B** Electron micrograph revealing ultrastructural features associated with hepatocytes, including epithelial cells lining a luminal structure arranged in one layer connected with tight junctions (circles) indicative of polarization. The surface facing the lumen contains numerous microvilli, whereas the abluminal surface facing the extracellular matrix remains smooth and attached to the underlying basal lamina (arrowheads). Scale bar 2 µm. **C** Immunohistochemical staining showing CK19-positive cells; the arrow denotes cholangiocytes surrounding the lumen. Scale bar is 200 µm. **D** Immunostaining of an organoid cryosection showing a later developmental cholangiocyte marker (CK7)-positive population of cells separate from the hepatocyte population. These form smaller ring-like structures as well as lining large luminal or cyst-like spaces within the organoids; scale bars are 100 µm. **E** Paraffin-embedded sections of organoids stained with alcian blue and counterstained with nuclear red. The lumen of this organoid shows cells containing pale blue cytoplasm, indicating the presence of mucopolysaccharides. Scale bar 50 µm. All of the above experiments were performed with the hiPSC line AG27.

Next, immunofluorescence was performed to corroborate both the scRNA-seq and proteomic findings of liver-like cellular complexity. Organoids (from Day 20 to 30) were stained against a battery of typical hepatocyte markers. We verified the presence of hepatocytes by the cytosolic expression of GS and CPS1 along with nuclear expression of the hepatic transcription factor HNF4α, which was located on the outer surface of the organoids (Fig. 4A and Supplementary Fig. 9). The enzymes GS and CPS1 are involved in the urea cycle and in adult liver and are zonally separated^46^. The process of zonation occurs throughout postnatal development, with GS being specifically absent from fetal liver hepatocytes^38^ and only detectable in the liver parenchyma after Day 2 post*partum* in humans^38^. The expression of GS in our organoids is thus indicative of a developmental stage surpassing that of fetal liver. This result is further reinforced by the expression of the xenobiotic metabolizing enzyme CYP2A6 (Fig. 4A and Supplementary Fig. 9), a bona fide marker distinguishing adult from fetal hepatocytes^47^, and the expression of ASGR1, a marker of maturity used to purify mature hepatocytes (Fig. 4A and Supplementary Fig. 9)^48^. To further validate a neonate stage, we assessed a panel of highly enriched adult liver genes and observed enrichment of this panel in our organoids (Supplementary Fig. 3).

Another feature of hepatocytes is polarization, which was first confirmed by the expression of the tight junction protein ZO-1 and the apical export protein MRP2, which are both enriched in bile canaliculi (Fig. 4A and Supplementary Fig. 9)^49,50^. Polarization was also assessed at the ultrastructural level, revealing features of primary liver tissue, including epithelial cells lining luminal structures arranged in a layer and connected by tight junctions, a feature of the hematobiliary barrier (Fig. 4B). Along with a polarized epithelial cell morphology with the lumen facing surfaces presenting with numerous microvilli, the abluminal surface facing the ECM was devoid of microvilli and appeared to be attached to an underlying basal lamina (Fig. 4B). The other endoderm-derived cell type, the cholangiocyte, is derived from the hepatoblast and lines the biliary ducts draining bile from the liver. We confirmed their presence in cells surrounding the lumen through immunohistochemical staining of CK19, which is present in cholangiocytes and hepatoblasts but is lost in hepatocytes (Fig. 4C), and CK7, which is expressed in vivo from 16 to 20 weeks postconception (wpc) in humans (Fig. 4D)^51^. Similar to the intestinal epithelium, biliary epithelial cells produce mucins to form a mucus layer. We explored whether we could detect the presence of mucus in our organoids. Using Alcian blue staining, we observed epithelial cells lining the lumen stained blue, indicating the presence of mucopolysaccharides, i.e., mucin-secreting cells (Fig. 4E). Interestingly, cholangiocytes express neutral and acidic mucins from 23–40 wpc^52^.

### iPSC-derived liver organoids are de novo vascularized

**Fig. 5:**
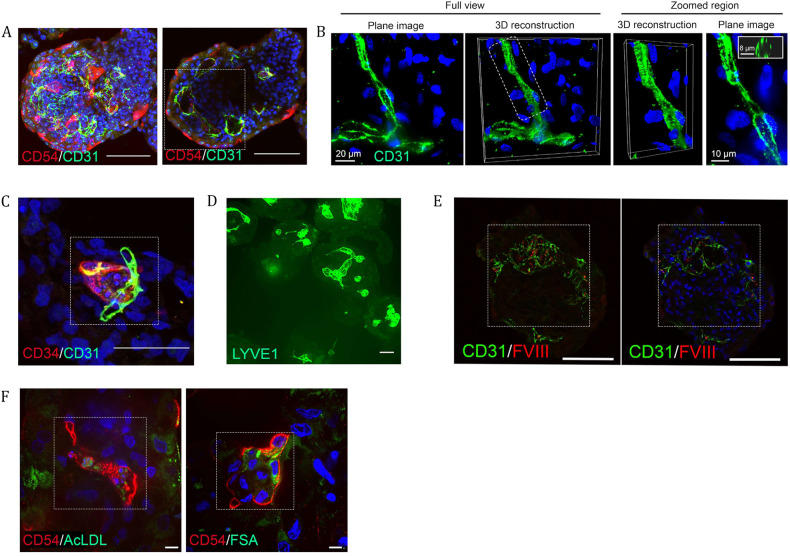
Liver organoids are de novo vascularized. **A** Max projection and cross section of whole mount immunostained organoids showing overlapping populations expressing one or both of the endothelial markers CD54 and CD31. Cross-section reveals stronger CD54 expression toward the outer surface contrasted with stronger CD31 expression toward the center of the organoid. Additionally, visible are multiple conjoined luminal spaces bounded by the positive cells. Scale bar, 100 μm; the highlighted area is magnified in Supplementary Fig. 9. **B** Immunofluorescence of CD31-expressing endothelial cells (green) under high magnification. Plane image and 3D volume rendering are shown. Hoechst 33342 (blue) dye was used to counterstain nuclei. Right panels show 3D volume rendering and a plane image of the selected region. Insert, visualization of vessel cross-section. **C** Immunostaining of a 50 μm cryosection showing adjacent and overlapping expression of CD34 and CD31 in a small structure indicative of neo-vascularization. Scale bar 50 μm, the highlighted area is magnified in Supplementary Fig. 9. **D** Whole mount staining of LYVE1, a sinusoidal endothelial marker, showing positive cells demarking a diversity of lumen shapes and sizes. Scale bar, 100 μm. **E** Immunostaining of two 50 μm thick cryosections showing co-localization of liver endothelial-associated FVIII and endothelial cell marker CD31 and luminal spaces in the organoids. Scale bar, 100 μm; the highlighted area is magnified in Supplementary Fig. 9. **F** Immunofluorescence analysis of AcLDL-488 and FSA-FITC binding and uptake in CD54^+^ endothelial cells within the organoids. Scale bar 10 μm, highlighted area is magnified in Supplementary Fig. 9. All of the above experiments were performed with the hiPSC line AG27 on organoids from Day 20 to Day 27.

Along with the parenchymal cells, the human liver is composed of many nonparenchymal cell types. We first investigated the endothelial populations identified by scRNAseq analysis. We used antibodies to delineate the different endothelial populations. We investigated the macrovasculature and observed branched chains and lumen surrounding structures positive for CD31 throughout the organoids (Fig. 5A and B and Supplementary Fig. 4a-c). To further validate the presence of vasculature within the organoids, we generated volume-rendered 3D reconstructions in cross-section, where we observed clear lumen with a median diameter of 8.3 μm (interquartile range of 6.9–10.7 μM), which is in the range of capillary lumens (5–10 μm) (Fig. 5B)^53^.

We observed small clusters of CD34^+^ endothelial structures suggesting continued neo-vascularization from approximately Day 20 and beyond and not just the expansion of earlier endothelial structures (Fig. 5C and Supplementary Fig. 9). In vivo, the microvasculature in the liver bud is acquired from the endothelial cells of the STM upon its invasion by hepatoblasts. These structures begin as CD34^+^/CD31^+^ continuous vasculature gradually acquiring liver sinusoidal endothelial cell (LSEC)-specific features. In the adult liver, LSECs exhibit distinct zonal markers^54^, which was recreated in the organoids where the endothelial structures acquire increasing CD54^+^ expression in proximity to the hepatocyte layer, which suggests that endothelial cell specialization is potentially a product of the niche (Fig. 5A and Supplementary Fig. 4b and Supplementary Fig. 9). We also investigated the distribution of LYVE1, another marker of LSECs that is associated with their scavenger function^55^, which revealed luminal structures within the organoids (Fig. 5D).

We next investigated endothelial cell functionality. We first examined Factor VIII, an essential component of the hemostatic system, which is expressed in the endothelial compartment, e.g., the sinusoidal endothelial cells of the liver in vivo^56^. To verify whether FVIII was expressed in the endothelial population of the organoids, we performed immunofluorescence, confirming the expression of FVIII protein in CD31-positive cells (Fig. 5E and Supplementary Fig. 9). The LSEC population is also equipped with high-affinity receptors (scavengers), enabling the removal of large molecules and nanoparticles from the blood to maintain blood and tissue homeostasis^57^. The proteomic data also indicated the presence of scavenger receptors (e.g., LYVE, LRP1, CD36 and SCARB1) in the organoids. LSECs can bind and take up acetylated low-density lipoprotein (AcLDL) and formaldehyde-treated serum albumin (FSA), which is linked to the scavenger receptor function^58^. Upon treatment of the organoids with AcLDL and FSA combined with immunofluorescence staining against the LSEC marker CD54^54^, we observed an association of both AcLDL and FSA with the CD54 LSEC population, indicating binding and uptake of these molecules (Fig. 5F and Supplementary Fig. 9).

**Fig. 6:**
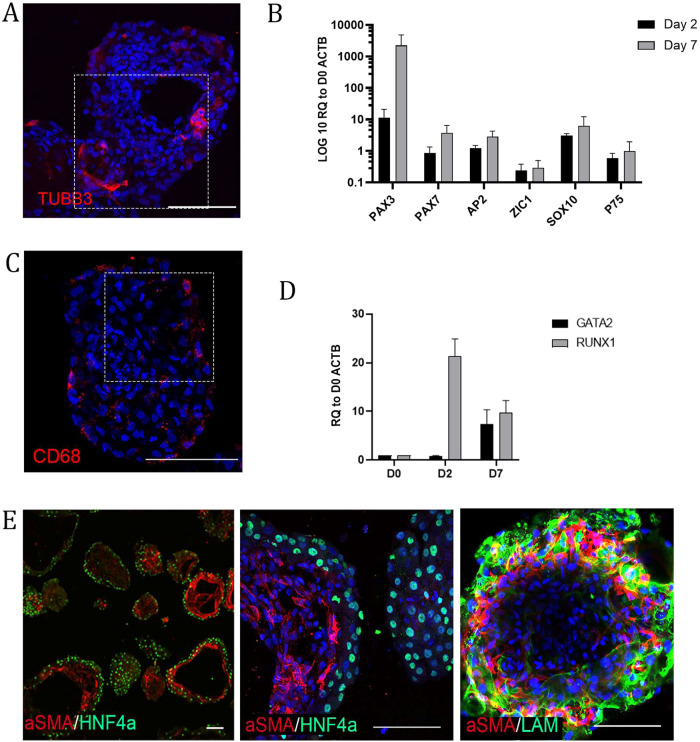
Liver organoids contain neuronal, resident macrophage and hepatic stellate populations. **A** Immunostaining for neuronal population in an organoid (TUBB3). The highlighted area is magnified in Supplementary Fig. 9. **B** RT‒qPCR analysis of neural crest stem cell markers at D2 and D7 of differentiation relative to D0 spheroids. The results from three independent experiments are presented as the mean ± SD. **C** Immunostaining of 50 μm thick cryosection showing CD68 in a granular pattern in the cytoplasm of the Kupffer-like cells. The highlighted area is magnified in Supplementary Fig. 9. **D** RT‒qPCR analysis of hemangioblast (*RUNX1*)- and hematopoietic (*GATA2*)-associated genes involved in the development of macrophages at D2 and D7 of differentiation relative to D0 spheroids. The results from three independent experiments are presented as the mean ± SD. **E** Immunostaining of cryosections showing expression of endodermal and stellate cell-associated proteins HNF4α and αSMA (left and middle panel) and (right panel) whole mount showing expression of mesenchymal and stellate cell-associated proteins (αSMA, Laminin) beneath the hepatocyte layer of organoids. All of the above experiments were performed with the hiPSC line AG27. All scale bars are 100 µm.

scRNAseq data identified a peripheral neuron population (Fig. 2A, G and H and Supplementary Fig. 2a, b and e). To corroborate these findings, we performed immunohistochemistry against TUBB3 (Fig. 6A and Supplementary Fig. 9), and we detected TUBB3-positive neurons throughout the organoids. Interestingly, the neural crest lineage arises from the pluripotent epiblast prior to definitive germ layer formation^44,45^. We investigated early points in the differentiation (Days 2 and 7) for the emergence of a neural crest-like population. Using RT‒qPCR, we observed the neural plate border markers *PAX3, PAX7*, and *ZIC1* and the neural crest specifiers *AP2* and *SOX10*. At Day 7, we observed the neural crest stem cell marker *p75* (Fig. 6B).

### iPSC-derived liver organoids contain a resident macrophage and hepatic stellate cell population

scRNAseq data identified a discrete population of Kupffer cells, which are the resident macrophage population of the liver and are derived in situ during the hematopoietic phase of liver development in vivo^59,60^. We investigated the presence of Kupffer cells within the organoids using the marker CD68^61^ and observed a CD68^+^ population exhibiting typical cytoplasmic granule staining (Fig. 6C and Supplementary Fig. 9). Endothelial and hematopoietic cells share a common precursor early in development called the hemangioblast. We speculated that the endothelial and Kupffer cell types potentially arise from a common mesodermal population at an early stage of differentiation. To that end, we assessed early time points in organoid differentiation using RT‒qPCR for orchestrators of hematopoietic commitment and observed the expression of *RUNX1* on Day 2, which is essential for hematopoietic commitment. By Day 7, we observed the expression of both *RUNX1* and *GATA2*, both players in the early hemangioblast core circuit (Fig. 6D)^62^. We speculate that part of this population will go on to form hematopoietic stem cells but are not yet specified to a myeloid lineage, from which Kupffer cells would ultimately emerge. Together, these data suggest that the mesoderm, by virtue of undergoing differentiation adjacent to endodermal-derived cells, facilitates the same role as the mesoderm in liver development in vivo. The scRNAseq analysis identified a putative hepatic stellate cell (HSC) population via canonical markers such as *BGN*, *CTGF*, *TPM2*, *SPARC*, *IGFBP*, *TAGLN*, *DCN*, *CCL2*, and *COL1A1*. In vivo, HSCs originate from the undifferentiated mesenchyme^32^. ALCAM^+^, MESP1^+^ and WT1^+^ populations were observed in the organoids (potentially a mesothelial/submesothelial equivalent) (Fig. 1H). We speculate that the WT1^+^ population potentially gives rise to an HSC population. We first assessed αSMA, revealing positive cells near the hepatocyte population, marked by HNF4α (Fig. 6E). HSCs with a star-like morphology that had long cytoplasmic processes with fine branches and cells resembling myofibroblasts were observed, suggesting quiescent and activated populations. This result is supported by the scRNAseq data, where both activated and resting populations were detected in UMAP space (Fig. 2A). We also cannot rule out that the HSCs are fetal in nature, as undifferentiated fetal HSCs express αSMA^63^. One function of HSCs is the production of ECMs, including laminins. Immunohistochemistry using a pan-laminin antibody along with αSMA revealed a close relationship between laminin and HSCs (Fig. 6E).

### iPSC-derived liver organoids display liver-like function

**Fig. 7:**
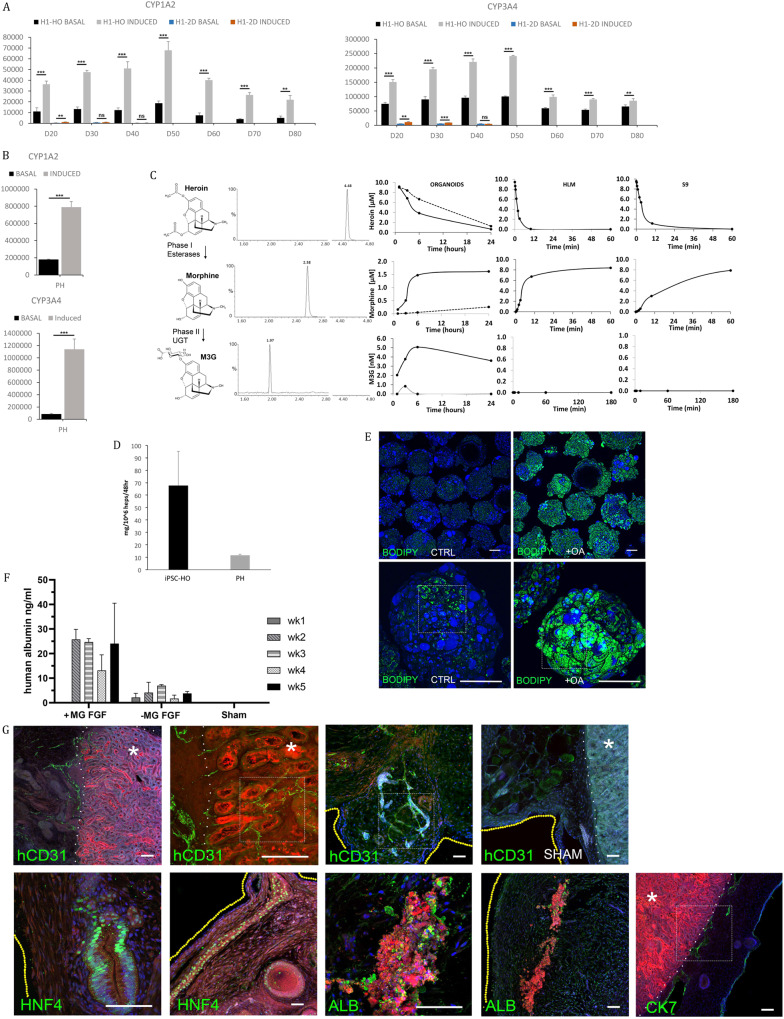
Assessment of liver organoid function, maintenance and transplantability. **A** Assay demonstrating inducibility and increasing activity of CYP1A2 (left side) and CYP3A4 (right side) drug metabolizing enzymes from D20 to D80 in the hESC line H1 organoids. For induction of CYP1A2, the cells were pretreated with omeprazole, and for CYP3A4, they were pretreated with rifampicin. Graphs show comparisons of suspension cultures and 2D differentiation. The results from three independent experiments are presented as the mean ± SD, ** *p* < 0.01, *** *p* < 0.001 with two-sided Student’s t test. **B** Assay demonstrating the activity and inducibility of CYP1A2 (top row) and CYP3A4 (bottom row) proteins in cryopreserved primary human hepatocytes. The results from three independent experiments are presented as the mean ± SD, ** *p* < 0.01, *** *p* < 0.001 with two-sided Student’s t test. **C** D20 AG27-derived organoids show phase I and phase II metabolism of heroin dosed at 10 μM. The left panel represents the metabolic pathway of heroin. Heroin is metabolized by sequential deacetylation (phase I reaction) to 6-monoacetylmorphine and morphine by esterase enzymes. Morphine is further glucuronidated (phase II reaction) by UDP-glucuronosyltransferases (UGTs) to morphine-3-glucuronide (M3G) and morphine-6-glucuronide (M6G). Center panel: Ion chromatograms of an extracted organoid sample analyzed by LC‒MSMS/MS. Right panel: Metabolism of heroin (10 µM) in organoids, primary human liver microsomes (HLMs) and the human S9 fraction (S9). **D** Demonstrating the production and secretion of urea into the culture medium from AG27-derived organoids (iPSC-HO) and primary human hepatocytes after 48 hours and normalized to mass per million hepatocytes (*n* = 3, mean ± SD, no urea was detectable in cell-free medium). **E** Whole-mount live imaging of oleic acid-treated (right column) and untreated (left column) AG27-derived organoids showing accumulation of nonpolar fats after treatment (BODIPY in green). Oleic acid was used at 300 μM for 5 days. The highlighted area is magnified in Supplementary Fig. 9. **F** Transplanted AG27-derived organoids can be maintained in mice. Human albumin was consistently detected over a 5-week period in mouse blood samples. D8 organoids were transplanted with either Matrigel/FGF2 supplementation ( + MG FGF) or Matrigel/FGF2 free (-MG FGF) (mean ± SD; +MG FGF *n* = 3, -MG FGF *n* = 2, sham *n* = 4). **G** Immunostaining of mouse kidney/organoid transplant cryosections demonstrates the retention of human hepatic populations (CK7, hCD31, HNF4α and ALB) and structures seen in vitro. They also demonstrated clear endothelial engraftment via hCD31 staining, whereas no hCD31-positive structures were visible in the sham group. Texas red dextran is strongly localized in the kidney parenchyma and albumin (ALB)-positive clusters of the transplanted organoids, while it is detectable at lower levels in other areas of the transplanted material. The boundary between the kidney parenchyma (marked with *) and organoid is delineated by a white dotted line, while a yellow dotted line marks the external surface. Nuclei are in blue, and all scale bars are 100 µm. Images that contain a white box have this area magnified and can be viewed in the Supplementary Fig. 9.

The organoids exhibit liver-like transcriptional and protein profiles, as well as a liver-like cellular repertoire. We next investigated their functional attributes. The liver has multiple functions, including the ability to metabolize drugs *via* CYP450 enzymes. We assessed the basal and induced levels of CYP1A2 and CYP3A4, which play an important role in the metabolism of a wide range of drugs, including caffeine and acetaminophen^64^. The organoids were benchmarked against primary human hepatocytes and a 2D protocol for generating hiPSC-derived hepatocytes^5,6^. The basal and induced CYP activity in 2D cultures was similar to previous reports (Day 20 of differentiation)^5,6^. The organoids presented with elevated levels of activity for both basal and inducible metabolism at the equivalent time point (Fig. 7A and B). We observed induction level differences of 5-fold for CYP3A4 and approximately 12-fold for CYP1A2 between organoids and primary hepatocytes. These levels of activity (basal and induction) were maintained over a 40-day period in the organoids, while activity rapidly declined to almost undetectable levels in 2D culture (Fig. 7A). We assessed long-term activity, observing maintenance of basal and inducible activity for 80 days (Fig. 7A). The activity declined from Day 50 for both CYP3A4 and CYP1A2; we speculate that this may be a feature of suboptimal culture conditions for long-term maintenance, possibly due to changing mass transfer conditions within the organoids (Fig. 7A).

The field of non-CYP450-mediated metabolism has attracted increasing attention as an important player in absorption, distribution, metabolism, and excretion (ADME)^65^. We investigated liver carboxyl esterases (CES), which are hydrolytic enzymes involved in the metabolism of endogenous esters, ester-containing drugs, pro-drugs and environmental toxicants^66^. The CES enzymes also metabolize a wide range of xenobiotic substrates, including heroin, which is metabolized by sequential deacetylation (phase I reaction) to 6-monoacetylmorphine (6-MAM) and morphine. Morphine is then glucuronidated by a phase II reaction via UDP-glucuronosyltransferase (UGT) to morphine-3-glucuronide (M3G) (Fig. 7C). We tested whether organoids supported heroin metabolism by exposure to 10 µM heroin and quantification by UHPLC‒MS/MS. We also compared organoid metabolism to human liver microsomes and the human S9 fraction (unfractionated microsomes and cytosol). The kinetics of metabolism were slower in the organoids, where we observed phase I metabolism of heroin by CES to morphine in approximately 6 hours, while the controls produced morphine in approximately 12 minutes. However, phase II metabolism (UGT) to morphine glucuronides was detectable in the organoids and absent in the controls (Fig. 7C).

Another essential liver function explored was hepatic urea synthesis, which is required for the removal of excess nitrogen. Above, we show the expression of CPS1 and GS enzymes, which are involved in the urea cycle (Fig. 4A). The organoids tested against a primary human hepatocyte control produced and secreted urea into the medium (Fig. 7D)^67^.

A study from Ouchi et al. (2019) demonstrated the accumulation of lipids in a liver organoid model, which displayed a steatohepatitis-like phenotype^25^. We tested whether the accumulation of lipids could be established in our organoids. Using the neutral lipid dye BODIPY, we established the steady-state level of lipids in untreated organoids, which was low (Fig. 7E (left panels) and Supplementary Fig. 9). After treatment with free fatty acids (oleic acid), the organoids showed substantial lipid accumulation in enlarged droplets (Fig. 7E (right panels) and Supplementary Fig. 9).

This ECM-independent system combined with small molecules can produce 300-500 organoids per ml of culture media, routinely producing tens to hundreds of thousands of organoids (see Supplementary Fig. 5a). Importantly, the cost of production has been reduced by nearly 3 orders of magnitude compared to that of conventional 2D approaches (Supplementary Fig. 5b). With access to large numbers of organoids, we assessed whether these could be transplanted and maintained in the kidney capsule of mice. The organoids were introduced into the kidney capsule either with ECM with fibroblast growth factor 2 (FGF2) or as pure organoids without ECM/FGF2. The rationale for the addition of FGF2 was that it has been reported that FGF2-containing Matrigel plugs promote neovascularization^68^. Using a human-specific albumin assay as a readout, we assessed the blood of mice over a 5-week period. No albumin was detectable in the first 96 hours; however, we observed secretion of human albumin into the bloodstream of the recipient mice from weeks 1 to 2 post-transplantation. This process was maintained until the mice were sacrificed at week 5 post-transplantation, while no human albumin was detectable in the control sham-treated mice (Fig. 7F). We then performed immunofluorescence staining on sections from the graft/host material to assess the presence and maintenance of hepatic cell types after transplantation. We demonstrate the retention of hepatic populations and structures that were observed in vitro. For example, we observed CK7-positive cells surrounding small and large luminal spaces within the transplanted material (Fig. 7G and Supplementary Fig. 9). Interestingly, we observed branching networks of human CD31-positive cells within the transplanted organoid that entered and extended throughout the mouse kidney parenchyma (Fig. 7G and Supplementary Fig. 9). We also observed the maintenance of both layers and luminal structures positive for HNF4α within the transplanted organoids (Fig. 7G). We also show cells highly positive for injected dextran within the mesenchyme area of the transplanted organoid, away from the kidney parenchyma. These cells were also positive for human albumin (Fig. 7G). The presence of human CD31 (human-specific antibody) vascular structures that appear to have anastomosed into the mouse kidney parenchyma further supports the presence of vascularization within our organoids and ongoing de novo vascularization.

### iPSC-derived liver organoids secrete plasma proteins and have functional coagulation machinery

**Fig. 8:**
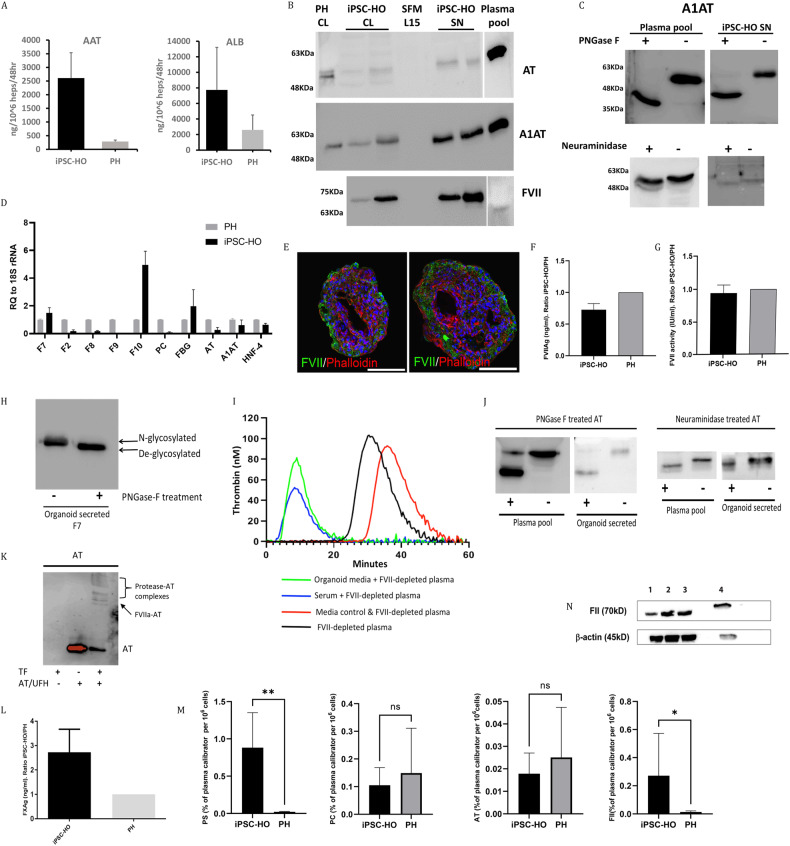
Assessment of liver organoid coagulation machinery. **A** Demonstration of the production and secretion of A1AT and albumin into the culture medium by D21-24 AG27-derived organoids and primary human hepatocytes over 48 hours as measured by ELISAs (*n* = 3, mean ± SD). **B** Antithrombin (AT), alpha-1-antitrypsin (A1AT) and factor VII (FVII) are present in cell lysates (Lanes 2 and 3) and supernatants (Lanes 5 and 6) derived from liver organoids assessed by SDS‒PAGE under reducing conditions. Plasma pool (Lane 7) and primary hepatocytes (Lane 1) were used as a reference; serum-free medium (SFM) L15 medium was used as a negative control (Lane 4). **C** Assessment of the N-glycosylation content of A1AT from the supernatant of organoids by PNGase F compared to the plasma pool. **D** Levels of coagulation factors and inhibitors in iPSC-derived organoids (black bars) and primary human hepatocytes (gray bars). mRNA levels of coagulation factors II, VII, VIII, IX, and X, fibrinogen (*F*2, *F*7, *F*8, *F*9, *F*10, *FBG*), the coagulation inhibitors protein C and antithrombin (*PC*, *AT*) and the hepatic markers alpha-1 antitrypsin and hepatocyte nuclear factor 4 alpha (*A1AT*, *HNF4α*) were determined using quantitative RT‒qPCR with 18 S as an endogenous control. The results are presented as the mean of the fold change expression of the respective gene. The results from three independent experiments are presented as the mean ± SD. **E** Immunostaining of two 50 μm cryosections showing localization of FVII (green) to the outer hepatocyte layer of the AG27-derived organoids, co-stained with phalloidin (red). Nuclei in blue, scale bars are 100 µm. **F** Demonstrating the production and secretion of FVII protein in the culture medium of AG27-derived liver organoids (iPSC-HO) and primary human hepatocytes (PH) as determined using ELISAs. The total concentration of FVII was adjusted to 1×10^6^ cells, and the results are expressed as the iPSC-HO/PH ratio. The results from three independent experiments are presented as the mean ± SEM. **G** Demonstrating FVII activity (IU/ml), culture medium from AG27-derived iPSC-HO and PH was determined using an FVII chromogenic assay. The results were adjusted to 1×10^6^ cells and are expressed as the iPSC-HO/PH ratio. The results from three independent experiments are presented as the mean ± SEM. **H** Assessment of the N-glycosylation content of secreted FVII of organoids by PNGase F treatment. **I** Assessment of thrombin generation in FVII-depleted plasma (black) with either serum-free medium (SFM) (red), organoid supernatant (green) or fetal bovine serum (FBS)-supplemented serum-free medium (SFM) L15 (blue). **J** Assessment of the N-glycosylation content of secreted AT from the supernatant of organoids by PNGase F and neuraminidase treatment compared to the human plasma pool. **K** Western blot analysis of AT after activation of organoid-derived supernatants by tissue factor (TF) and CaCl_2_ and incubation with AT and unfractionated heparin. Activated FVII-AT (FVIIa-AT) complexes are indicated by an arrow and bracket. l Left panel: FX protein (Ag) levels (ng/ml) in culture medium from iPSC-HO and PH as measured by ELISAs. The total concentration of FX was adjusted to 1×10^6^ cells, and the results are expressed as the iPSC-HO/PH ratio. The results from three independent experiments are presented as the mean ± SEM. **M** Demonstrating the production and secretion of PS, PC, AT and FII proteins in the culture medium of AG27-derived liver organoids (iPSC-HO) and primary human hepatocytes (PH) as determined using the Procarta-plex assay. The total concentration of AT was adjusted to 1×10^6^ cells, and the results are expressed as the percentage of the plasma calibrator control. The results from three independent experiments are presented as the mean ± SD. Statistical significance was assessed with the Mann‒Whitney test (*p < 0.05, **p < 0.01). **N** Intracellular levels of prothrombin (FII) shown by western blots, lysates from iPSC-HO (Lanes 1-3) and PH (Lane 4). Equal amounts of proteins were separated by SDS‒PAGE under reducing conditions. β-Actin was used as a loading control. The results of three independent experiments are presented.

An important feature of the liver is the production of a plethora of serum proteins, which is estimated at nearly 700^69^. These include the major plasma proteins, apolipoproteins, coagulation factors and hormones. We have demonstrated the production of HGF from our organoids (Fig. 1M); upon inspecting the proteomic datasets, we identified numerous serum proteins, including apolipoproteins (APOA1, APOA4, APOC3 and APOD), hormones (the IGFs) and serine protease inhibitors (A1AT, AT, C1-inhibitor, etc.) (Fig. 3A). We next confirmed the production and secretion of several hepatic proteins. We first assessed the production/secretion of albumin and A1AT by ELISAs, as these molecules were previously shown to be transcribed (Fig. 1K) and expressed above (albumin) in hepatocytes (Fig. 1L). Both of these proteins are secreted from hepatocytes into the circulation in vivo, and their secretion into the culture medium was also verified (Fig. 8A). We assessed A1AT in both the supernatants and lysates of organoids by WB analysis. Notably, the A1AT electrophoretic mobility was very similar to that of those forms detected in both human plasma and primary hepatocytes (PH) (Fig. 8B). A1AT contains several PTMs that increase protein stability, for example, protecting against proteolysis and degradation, among other things^70,71^. We analyzed the N-glycosylation status of A1AT, which has four known N-glycosylation sites^72^. N-glycosylation status was investigated by treatment with PNGase F and neuraminidase. We showed a higher electrophoretic mobility under both treatments, compatible with the removal of 4-glycans and sialic acid groups on A1AT (Fig. 8C). These results were similar to those observed in the control plasma pool, where similar shifts in mobility were observed (Fig. 8C).

Another key function of the liver is the production and secretion of coagulation factors and inhibitors that maintain balanced hemostasis. We investigated whether we could detect the expression of vitamin K-dependent coagulation factors, endogenous anticoagulants, and a number of other coagulation factors by RT‒qPCR. The expression of vitamin K-dependent coagulation factors (except for *F9*, which exhibited very low expression), the two main natural hepatic anticoagulants (protein C (*PC*) and *AT*), *F8* (expressed in endothelial cells), and plasma fibrinogen (*FBG*) were all observed (Fig. 8D). Interestingly, the mRNA levels of *F10* showed elevated expression (Fig. 8D) compared to those of the other coagulation factors. We speculate that the elevated levels may be a feature of the activated HSC population, which (indicative of fibrosis) can lead to elevated *F10*^73^ and is worthy of further investigation.

We are particularly interested in FVII and its associated machinery and the development of effective coagulation cell-based models, which are currently lacking in this space. We first assessed FVII by immunofluorescence and observed that this procoagulant molecule was localized to the outer epithelial cells, corresponding to the hepatocytes (Fig. 8E). We assessed the production and secretion of FVII into the supernatant (media) by WB, observing similar electrophoretic mobility in both the lysate and secreted compartments (Fig. 8B). Next, we used ELISAs, demonstrating robust production of FVII at comparable levels to primary human hepatocytes (Fig. 8F). As FVII has a key role in the initiation of blood coagulation, the activity of FVII was assessed using a chromogenic assay, where we observed levels comparable to primary human hepatocytes (Fig. 8g). FVII contains PTMs; notably, it undergoes N-glycosylation, which is important for its secretion. We investigated the N-glycosylation status of secreted FVII by PNGase F digestion, which showed a higher electrophoretic mobility compatible with the loss of 2 N-glycans (Fig. 8H). We then evaluated the ability of the secreted FVII (and the other coagulation factors) to generate thrombin using a thrombin generation assay. The cell medium from the hepatic organoids shortened the lag time of FVII-deficient plasma when mixed at equal volume with FVII-deficient plasma (Fig. 8I).

We next assessed the production of other components of the coagulation machinery, including AT, which is one of the most potent anticoagulant proteins that inhibits multiple coagulation serine proteases, mainly thrombin (FIIa) and activated factor X (FXa) but also FVIIa, FIXa, FXIa and FXIIa. WB analysis showed that intracellular and secreted AT from organoids had similar electrophoretic mobility to primary hepatocytes and human plasma AT (Fig. 8B). We investigated the PTMs of AT using PNGase F and neuraminidase-based digestion. After treatment, a higher electrophoretic mobility was observed, which was similar to the plasma pool control and compatible with 4-glycan removal (Fig. 8J). Additionally, mobility changes were observed after neuraminidase digestion, consistent with the loss of 8 terminal sialic acids associated with the N-glycans (Fig. 8J), similar to those observed with the human plasma pool (Fig. 8J). The functionality of AT produced by organoids was tested by the detection of covalently bound thrombin-AT complexes, which were generated by the addition of thromboplastin (TF + ) to iPSC-HO supernatants. The analysis also detected FVII-AT complexes (Fig. 8K). We also evaluated the expression of procoagulant proteases that are targets of AT. Secretion of FX was detected using ELISAs (Fig. 8L), while PS, PC, AT and FII were quantified using Procarta-plex assays (Fig. 8M). Finally, we showed the intracellular levels of prothrombin (FII) by western blotting (Fig. 8N).

### Organoids can be derived from multiple pluripotent stem cell lines

Another caveat for a robust differentiation protocol is the ability to apply it to multiple lines; to that end, we assessed the differentiation of 2 additional pluripotent stem cell lines, 207 and H1 (Supplementary Fig. 6a). In all cases, the differentiation proceeded as described above. We observed the progression from pluripotency to mesoderm and DE; and *T*, *GSC*, *FOXA2*, *SOX17*, *HHEX* and *CER1* (Supplementary Fig. 6a and b); then, the mesendodermal aggregates were hepatically specified for 5 days, resulting in the expression of *AFP*, *CEBPa*, *HNF4α, PROX1*, *TBX3*, and *TTR* coinciding with decreased expression of DE markers such as *HHEX*, *SOX17* and *GATA4* (Supplementary Fig. 6c). Next, over a 14-day period, the early hepatic organoids were directed to a mature liver-like stage. The resulting organoids exhibited markers of hepatic maturation and tissue-like cellular complexity. RT‒qPCR revealed expression of the hepatic markers *A1AT*, AFP, *ALB*, and *ASGR1* as well as genes enriched in the liver such as *APOA2*, *TDO2* and *TTR* and xenobiotic metabolism, cytochrome P450 (*CYP) 3A4* and *CYP3A7* (Supplementary Fig. 6d). The organoids exhibited key liver functions, such as serum protein production (Supplementary Fig. 6e and f) and urea production (Supplementary Fig. 6g). They also exhibited CYP450 activity (Supplementary Fig. 6h and i). We further characterized the organoids by imaging against a panel of hepatic markers for hepatocytes (HNF4α and ASGR1), macrophages (Kupffer cells) (CD68), cholangiocytes (CK7), hepatic stellate cells (αSMA) and vasculature (CD31), where all markers were represented (Supplementary Fig. 7a and b). We also assessed the organoids for their ability to accumulate lipids after treatment with oleic acid for 5 days, with imaging showing large accumulation of lipids (Supplementary Fig. 7c and d). We also assessed the production of coagulation factors using Procarta-plex assays. We showed the secretion of PS, PC, AT and FII into the media (Supplementary Fig. 8a and b). In addition, the organoids produced and secreted Factor VII (Supplementary Fig. 8c). Finally, we performed RT‒qPCR against the same coagulation machinery panel described in Fig. 8D and observed similar profiles (Supplementary Fig. 8d).

## Discussion

Scalable production of liver-like tissue that exhibits functionally long-lived human liver characteristics has remained elusive. There have been efforts to produce large amounts of liver organoids, for example, a study by Schneeberger and colleagues^74^ using LGR5+ adult stem cells. These researchers produced organoids composed exclusively of hepatocytes for transplantation purposes, thus limiting their utility. Additionally, this protocol is dependent on supplementing the media with 10% Matrigel and expensive growth factors. Another approach used by Takebe et al. was the massive production of liver buds^75^, but this method had the limitation of requiring transplantation to drive maturity. Here, we describe an approach that can generate substantial amounts of functionally mature hPSC-derived liver-like organoids. The described protocol is straightforward, efficient, reproducible, and applied to different lines, and organoids can be produced in just 20 days. The organoid differentiation protocol follows a liver-like developmental route, producing organoids that contain a liver-like cellular repertoire including the parenchymal hepatocytes and cholangiocytes. These organoids also contain nonparenchymal cells, hepatic stellate cells, Kupffer cells and endothelial populations. Interestingly, further analysis by scRNAseq indicated the establishment of early hepatocyte zonation. The process of liver zonation and its establishment are still not fully understood. It is becoming apparent that liver zonation in humans takes many years to be established. Liang et al.^42^ demonstrated that in mice, hepatocyte zonation only became defined after Day 56 post-birth. Only then could the researchers clearly distinguish identity based on spatial locations, and differential expression of zonation markers was observable. This finding demonstrates that zonation in the case of mice takes many weeks to be established and extrapolating to humans, months to years. In the case of our organoids, we observed evidence of the emergence of partial hepatocyte zonal identity, albeit not perfect, and as highlighted in Liang et al.^42^, the zonation profile is progressively built up during postnatal liver development. In the case of LSEC zonation, we did not detect significant differential expression of LSEC markers or gene signatures in the EC clusters, indicating that zonation is not yet established in our model. This finding is not unexpected; one only observes pericentral and periportal LSEC populations after Day 56 in rodents^42^, equivalent to many months to years in humans. This process can be compared to the establishment of ploidy in humans, as all hepatocytes are diploid in neonates and approximately 40% of hepatocytes are polyploidy by late teens, these processes all take time.

Proteomic analysis also clearly demonstrated a liver-like phenotype. scRNAseq data were further confirmed using immunostaining, where we observed the aforementioned cell types. Interestingly, we observed parenchymal cell polarity, which was supported by electron microscopy. We also confirmed the presence of nonparenchymal cell types, including stellate and Kupffer cells. Remarkably, we observed de novo vascularization within our organoids. The organoids showed the presence of continuous luminal structures, reconstructed in 3D, with a diameter of 8 μm. The organoids presented with liver-like functional features, which included the production of serum proteins and coagulation factors and supported ureagenesis. They are proficient in drug metabolism exhibiting long-term activity, 80 days, with respect to CYP metabolism. They also exhibited non-CYP-mediated metabolism using heroin as an exemplar. The organoids can accumulate fatty acids, presenting with a steatotic-like phenotype, potentially providing a useful model for nonalcoholic fatty liver disease (NAFLD). The organoids can be successfully transplanted into mice, where they stably produce human albumin. The engrafted organoids show human CD31-positive endothelial structures throughout large areas of the transplanted organoid, extending into the kidney parenchyma of the mouse. This process could only occur through vascularization from the organoids into the surrounding mouse tissue, supporting our claims of de novo vascularization.

With respect to disease modeling and normal cellular processes, an area we are currently pursuing is the utility of our liver organoids to model the coagulation machinery. We lack suitable cellular models that can recapitulate key features of this process and produce physiological and correct PTMed proteins. Access to patient-specific liver organoids harboring coagulation factor deficiencies would allow access to autologous liver cells that can be exploited to elucidate complex genetic defects that cannot currently be detected using patient plasma. We have demonstrated that the organoids are able to produce AT, A1AT, and high levels of FVII and other vitamin K-specific factors, including FII and FX. Moreover, the electrophoretic mobility of the intracellular and extracellular forms of the organoid-derived proteins was very similar to their plasma equivalents of AT, A1AT and FVII. The organoid-derived proteins AT, A1AT and FVII were N-glycosylated, compatible with 4, 4 and 2 mature N-glycans, respectively, and similar to those found in the plasma of healthy adults. Finally, the above approach to evaluate organoid-derived FVII function suggests that although these experiments are not specific for FVII activity per se, supernatants (supplemented with vitamin K) show hemostatic potential due to the secretion of serine proteases (probably gamma-carboxylated) into the conditioned medium that mimic the FVII effect.

Organoids provide both a unique and powerful model to interrogate disease, understand regeneration and potentially provide the building blocks for bridging therapies for patients waiting for an organ or ultimately provide a replacement organ. A key limitation is scaling; for example, in children, 10^9^ hepatocytes are required to correct specific metabolic liver function^76^; however, researchers are currently producing organoids in the 10 s to 100 s in combination with nondefined Matrigel (ECM) and recombinant growth factors, making scaling both a financial and technical bottleneck. Our approach currently produces up to 500 organoids per ml (Supplementary Fig. 5a) in Erlenmeyer flasks. Based on our approach, we would require culture volumes of 1–3 liters to achieve these numbers. The approach is also compatible with the controlled production in stirred tank bioreactors as recently demonstrated for cardiac and hematopoietic lineages^77,78^ and will provide the field with a potentially game-changing resource to allow the development of clinical as well as screening platforms where the requirement will be in the millions of organoids rather than today’s laboratory scale.

More work is required to investigate the potential of these scalable organoids in the context of transplantation for bridging therapies but will potentially provide an important treatment modality for non-reversible acute liver failure^79^. Timing of the transplantation is critical for acute liver failure, and many patients receive a graft from a marginal donor instead of waiting for a better offer, which is associated with worse post-transplant outcome^80,81^, or succumb to disease without transplantation. Therefore, approaches that can improve the condition and prolong the survival of patients with acute liver failure awaiting a liver transplant would increase the number of potential organ offers for a cadaveric graft, reducing the use of marginal donors and even allowing transplants to be performed in patients currently not receiving a graft offer. We envisage that these organoids will provide a powerful tool to address developmental biology in the dish, where we could utilize lineage tracing/barcoding to investigate the emergence of, for example, HSCs and tissue-resident hematopoietic cells, i.e., the Kupffer population. In addition, these organoids will provide a powerful tool to allow the investigation of cellular interactions, i.e., the mesoderm and endoderm, and how they contribute to inducing or co-developing these lineages. Organoid production is scalable at a cost-effective level but will require standardization to provide a platform for drug screening, toxicology, and disease modeling or to act as building blocks to produce liver microtissues and potentially scale to larger tissue units.

### Supplementary information

Supplementary Information

Supplementary Information Table 1 dataset
