# Supplementary material for: Scalable production of tissue-like vascularized liver organoids from human PSCs
Source: Exp Mol Med. 2023 Sep 1;55(9):2005–24. doi: 10.1038/s12276-023-01074-1 (PMC10545717; doi:10.1038/s12276-023-01074-1)
Supplement: Supplementary file 1 — Supplementary Information [file 12276_2023_1074_MOESM1_ESM.pdf]

Title: Scalable production of tissue-like vascularised liver organoids from human PSCs.

Running Title: Scalable liver organoid production.

Authors: Sean P Harrison[1,2†], Richard Siller[3†], Yoshiaki Tanaka[4,5†], Maria E Chollet[6,7†], María Eugenia de la Morena-Barrio[8], Yangfei Xiang[4], Benjamin Patterson[4], Elisabeth Andersen[6,7], Carlos Bravo-Pérez[8], Henning Kempf[9], Kathrine S Åsrud[10,11], Oleg Lunov[12], Alexandr Dejneka[12], Marie-Christine Mowinckel[6,7], Benedicte Stavik[6,7], Per Morten Sandset[6,7,11], Espen Melum[1,7,10,11,13,14], Saphira Baumgarten[1,2], Flavio Bonanini[15], Dorota Kurek[15], Santosh Mathapati[3], Runar Almaas[2,11,14], Kulbhushan Sharma[3], Steven R Wilson[1,16], Frøydis S Skottvoll[1,16], Ida C Boger[16], Inger L Bogen[17], Tuula A Nyman[18], Jun Jie Wu[19], Ales Bezrouk[20], Dana Cizkova[21], Javier Corral[8], Jaroslav Mokry[21], Robert Zweigerdt[9], In-Hyun Park[4], Gareth J Sullivan[1,2,3,18*]

Supplementary Materials:

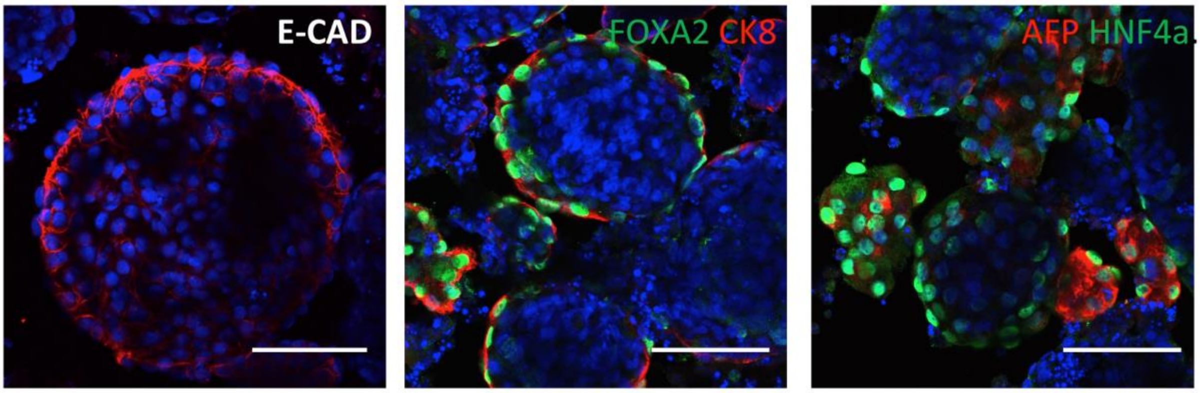

<u>**Supplementary Figure 1.**</u> **Organisation of D7 AG27 derived organoids**. Whole-mount immunostaining showing a cross section through the D7 organoids showing the restriction of epithelial (ECAD) and early hepatocyte markers (FOXA2, CK8, HNF4α, AFP) to the outer surface of the organoids and their absence from the inner core. Nuclei stained with DRAQ5, scale bars are 100 µm.

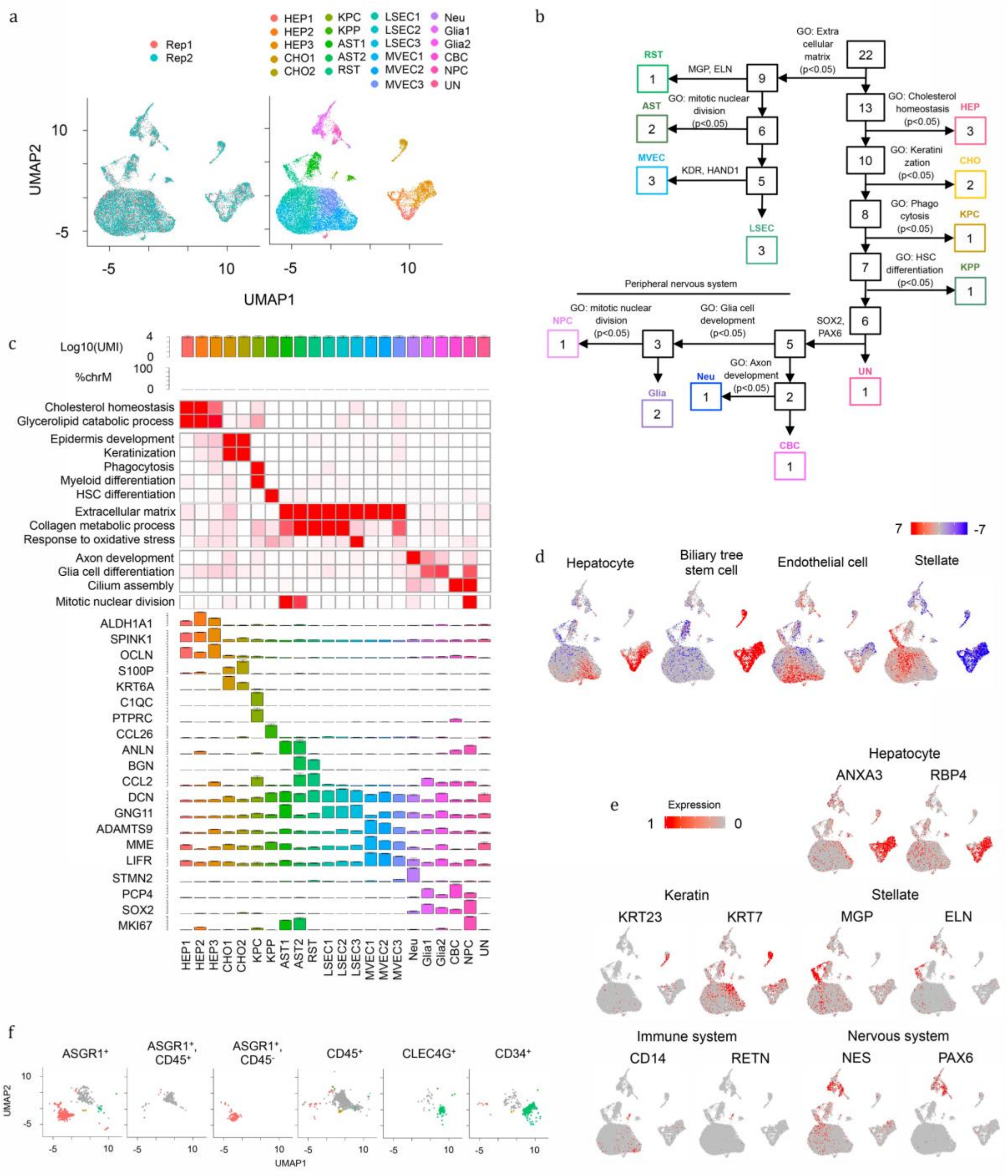

Supplementary Figure 2. Cell Type Characterization in Single-Cell Transcriptome Profiles. (a) UMAP plot of single cells distinguished by replicates and clusters. (b) Schematic representation of cluster annotation method. (c) Comparison of total UMI, percentage of mitochondria-derived reads, GO enrichment, representative gene markers across 22 clusters. (d) GSEA of gene signatures of hepatocyte, biliary tree stem cell, endothelial cell and stellate. The enrichment and depletion are scaled by -log10(FDR) and shown by red and blue colors, respectively. (e) Expression pattern of cell-type specific markers in the liver organoid. Relative expression level is plotted from gray to red colors. (f) UMAP plots of FACS-sorted cells from human livers. Color scheme and UMAP dimension correspond to Fig. 2e. All above experiments were performed with the hiPSC line AG27.

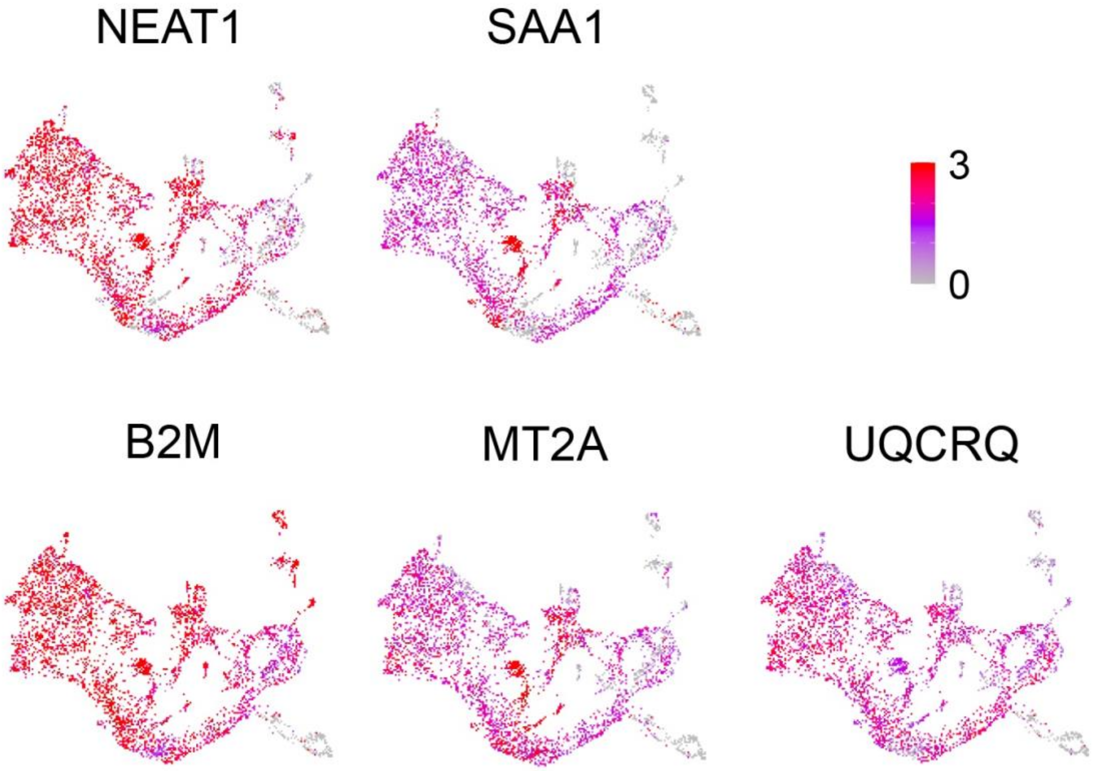

<u>Supplementary Figure 3.</u> Analysis of scRNAseq organoid data vs. Adult Liver. The analysis highlights representative genes that are highly expressed in adult hepatocyte and AG27 derived organoids. The colour represents gene expression (normalized RNA-seq read count).

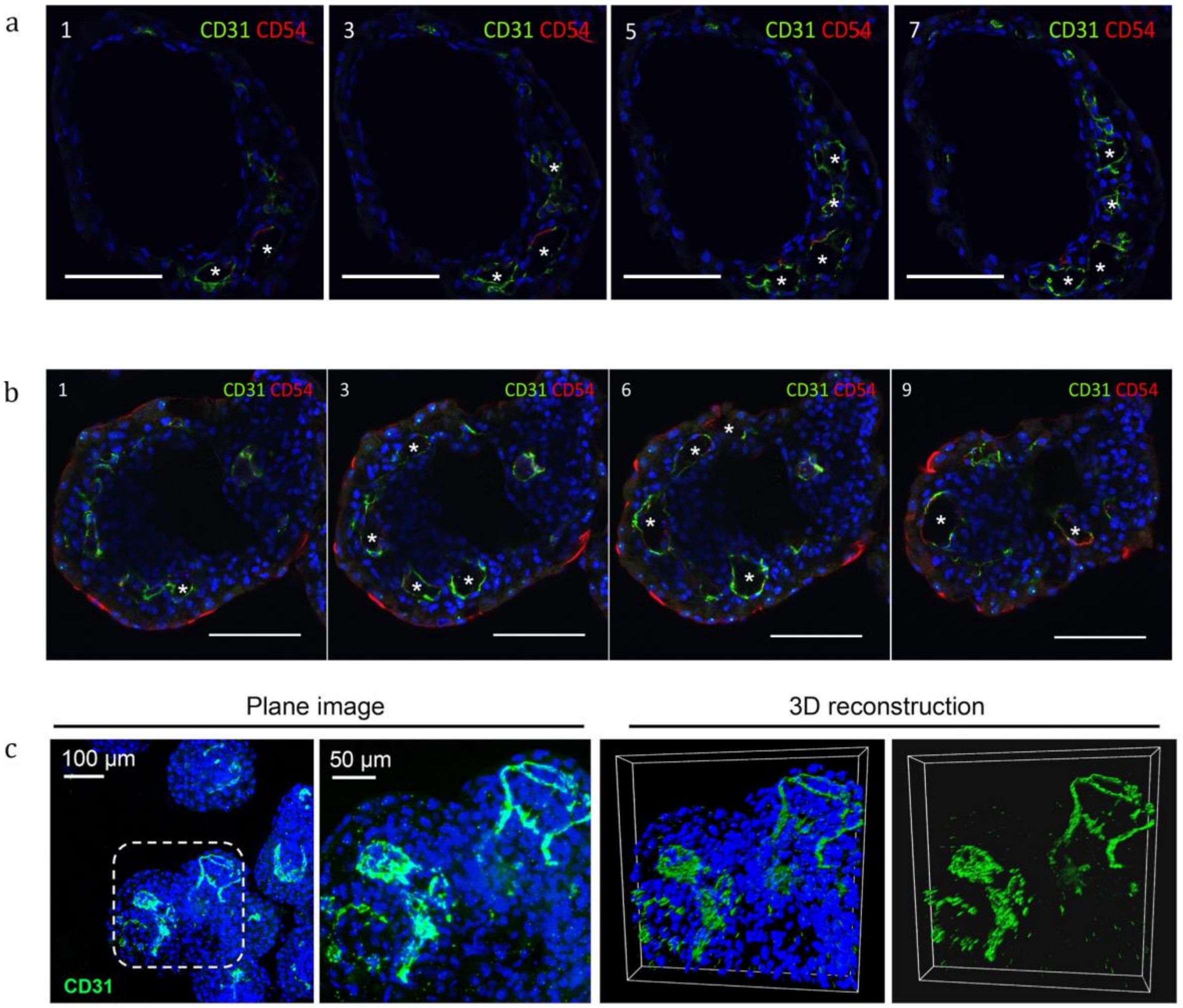

<u>Supplementary Figure 4.</u> Endothelial structures in D20-D30 Organoids. (a) and (b) z planes covering a volume of 35 µm (a) or 45 µm (b) from cryosections immunostained (same organoid as Fig. 5A) for the endothelial markers CD31 and CD54 showing continuity of luminal spaces and changes in endothelial marker expression towards the outer surface of the organoids. Number in top left (in white) represents position in the z volume, all Scale bars are 100 µm. (c) Immunofluorescence of CD31-expressing endothelial cells (green) shows establishment of vascular networks in organoids. With zoomed area in hatched white box, Hoechst 33342 (blue) dye was used to counterstain nuclei. Confocal 3D volume rendering of the organoid is presented on right panels, showing organoid structure both with nuclei and vascular networks only.

a

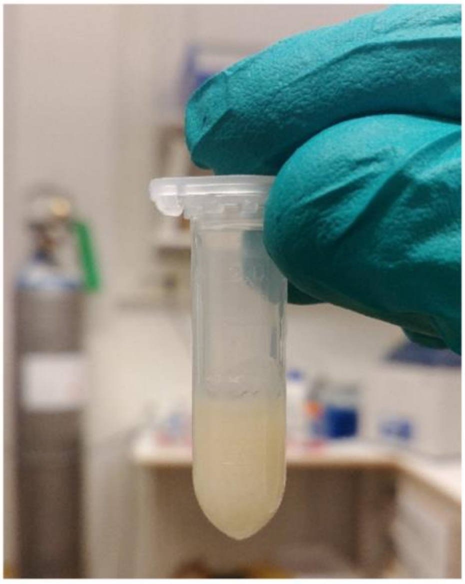

b

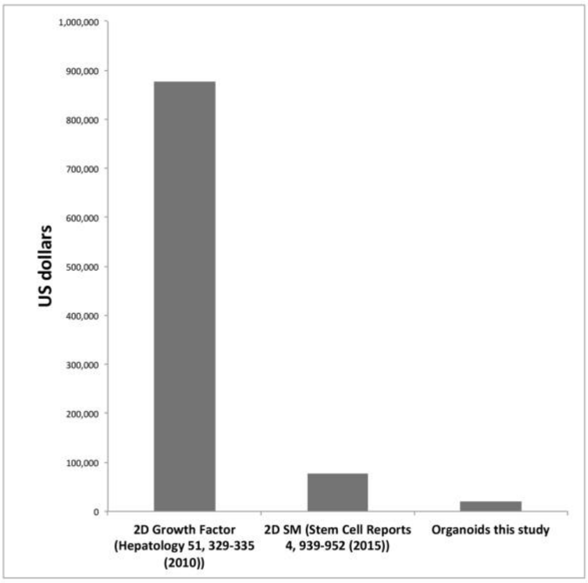

Supplementary Figure 5. A Scalable Culture System. (a) Image showing pellet of end stage organoids from 35 ml of suspension culture after centrifugation, approximately 18,000 organoids in a 700 µl packed pellet. (b) Cost comparison in US dollars for the production of approximately $2x10^{10}$ cells based on a previously published methods using growth factor in 2D [6], a small molecule-based differentiation [8] and this study.

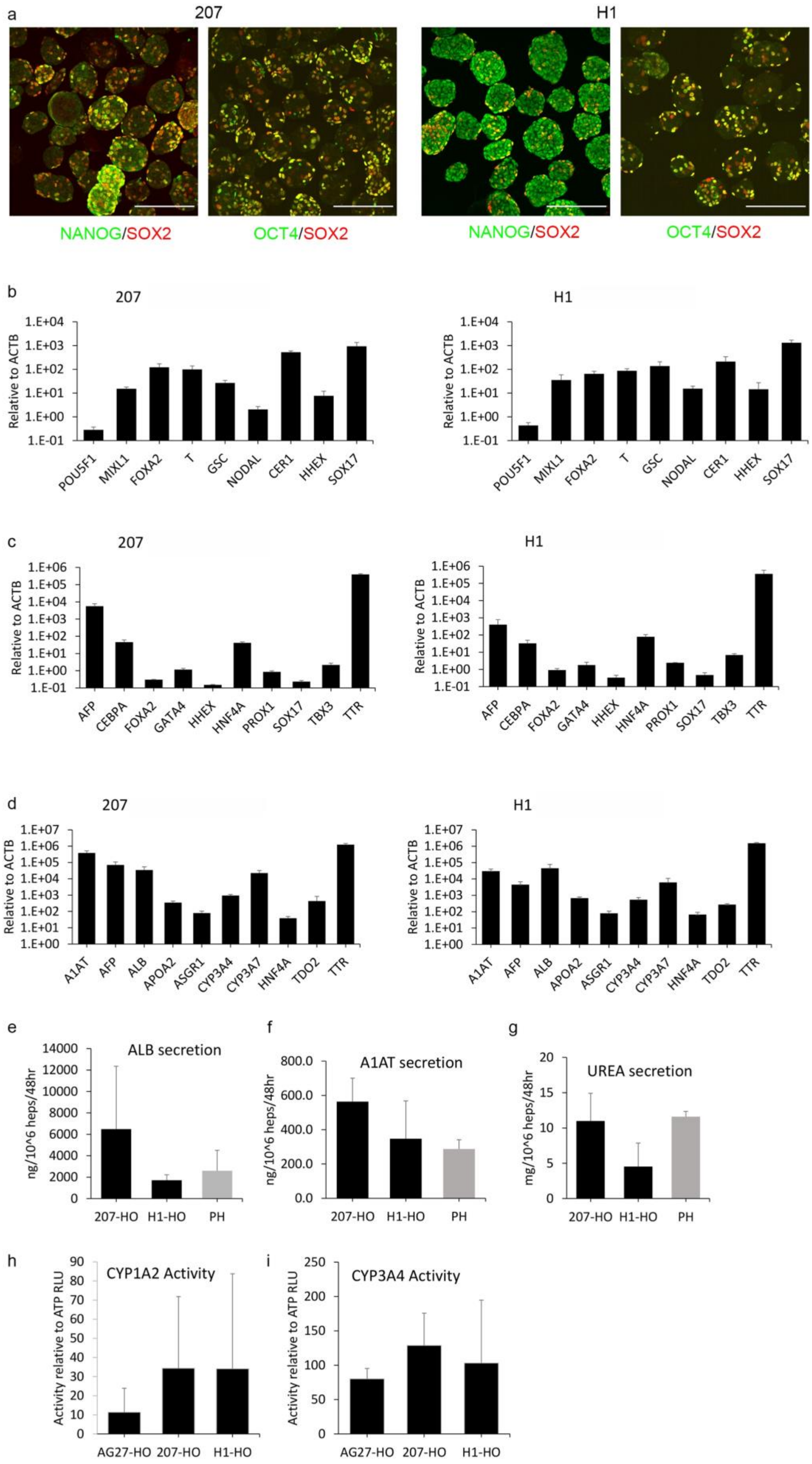
a
207
H1
NANOG/SOX2
OCT4/SOX2
NANOG/SOX2
OCT4/SOX2
b
207
H1
Relative to ACTB
POU5F1
MIXL1
FOXA2
T
GSC
NODAL
CER1
HHEX
SOX17
c
207
H1
AFP
CEBPA
FOXA2
GATA4
HHEX
HNF4A
PROX1
SOX17
TBX3
TTR
d
207
H1
A1AT
AFP
ALB
APOA2
ASGR1
CYP3A4
CYP3A7
HNF4A
TDO2
TTR
e
ALB secretion
ng/10^6 heps/48hr
f
A1AT secretion
ng/10^6 heps/48hr
g
UREA secretion
mg/10^6 heps/48hr
207-HO
H1-HO
PH
h
CYP1A2 Activity
Activity relative to ATP RLU
i
CYP3A4 Activity
Activity relative to ATP RLU
AG27-HO
207-HO
H1-HO

Supplementary Figure 6. Characterising hESC (H1 and 207) derived organoids. (a) Representative images of Day 0 (D0) pluripotent spheroids derived from the hESC lines 207 and H1. Whole-mount immunostaining of the pluripotency markers OCT4, SOX2 and NANOG, scale bar is 200 µm. (b) RT-qPCR analysis of pluripotency and DE associated genes at D2 of the differentiation relative to D0 spheroids on a log10 scale, in hESC lines 207 and H1. Results from three independent experiments are presented as mean ±SD. (c) RT-qPCR analysis of DE and early liver development genes at D7 of the differentiation relative to D2 on a log10 scale, in hESC lines 207 and H1. Results from three independent experiments are presented as mean ±SD. (d) RT-qPCR analysis of both early and later (developmentally) hepatocyte genes at D21 of the differentiation relative to D2 on a log10 scale, in hESC lines 207 and H1. Results from three independent experiments are presented as mean ±SD. (e) Demonstrating the production and secretion of albumin into the culture medium by D21 hESC lines 207 and H1 derived organoids (HO) and primary human hepatocytes (PH) after 48 hours as measured by ELISA (n=3, mean ±SD) and normalized to mass per million hepatocytes. (f) Demonstrating the production and secretion of A1AT into the culture medium by D21 hESC lines 207 and H1 derived organoids and primary human hepatocytes after 48 hours as measured by ELISA (n=3, mean ±SD) and normalized to mass per million hepatocytes. (g) Demonstrating the production and secretion of urea into the culture medium from 207 and H1 derived organoids and primary human hepatocytes after 48 hours and normalized to mass per million hepatocytes (n=3, mean ±SD). (h) Assay comparing activity of CYP1A2 drug metabolizing enzyme from D23 to D30 in the PSC derived AG27, 207 and H1 organoids. Values were normalized to ATP assay performed on the same wells. Results from three independent experiments are presented as mean ±SD. (i) Assay comparing activity of CYP3A4 drug metabolizing enzyme from D23 to D30 in the PSC derived AG27, 207 and H1 organoids. Values were normalized to ATP assay performed on the same wells. Results from three independent experiments are presented as mean ±SD.

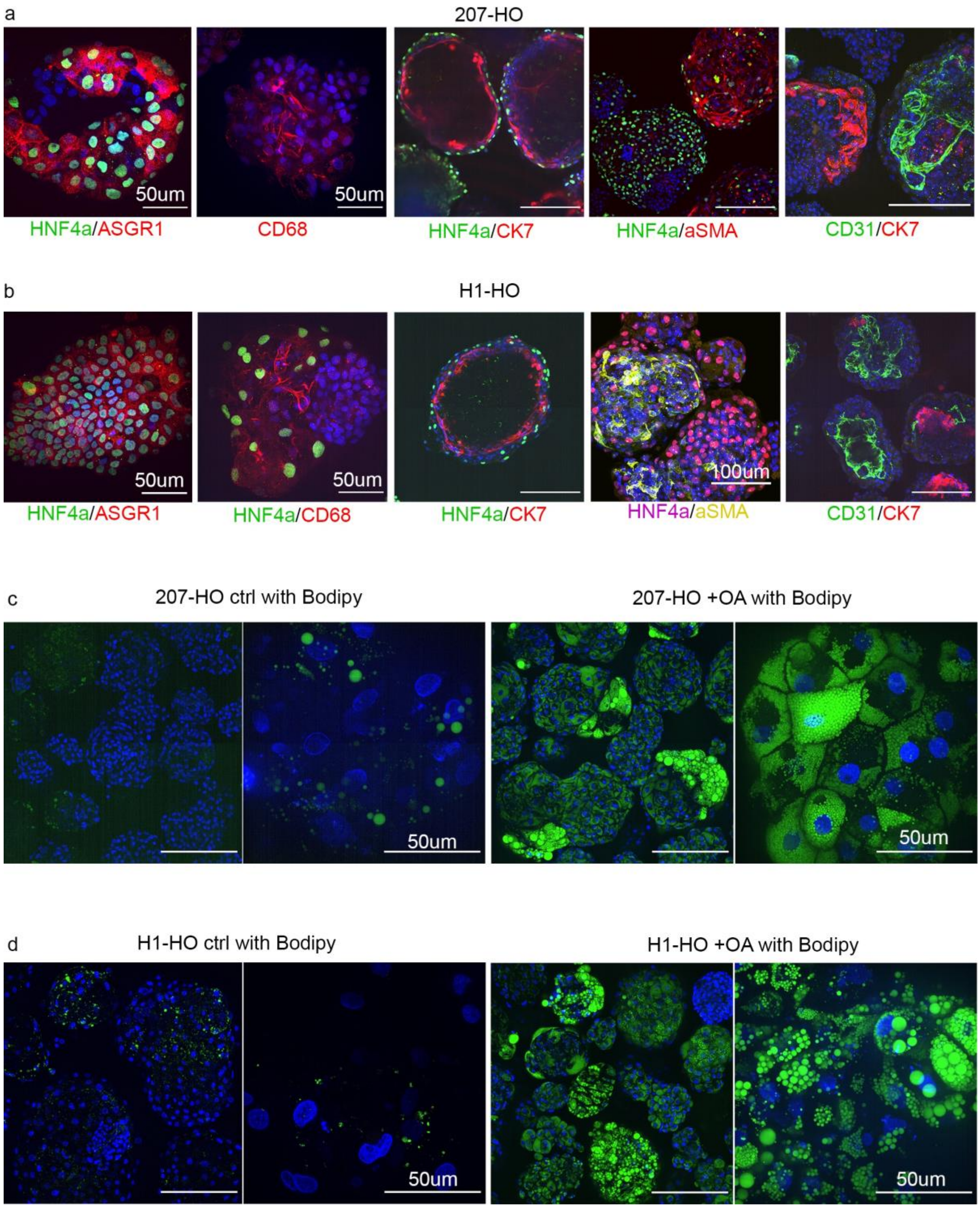

a
207-HO
50um
50um
HNF4a/ASGR1
CD68
HNF4a/CK7
HNF4a/aSMA
CD31/CK7
b
H1-HO
50um
50um
100um
HNF4a/ASGR1
HNF4a/CD68
HNF4a/CK7
HNF4a/aSMA
CD31/CK7
c
207-HO ctrl with Bodipy
207-HO +OA with Bodipy
50um
50um
d
H1-HO ctrl with Bodipy
H1-HO +OA with Bodipy
50um
50um

<u>Supplementary Figure 7. Characterising hESC (H1 and 207) derived organoids.</u> (a) Wholemount immunostaining showing expression of various markers of liver cell types found within the organoids derived from hESC line 207. Showing hepatocyte markers (HNF4a and ASGR1), macrophage marker (CD68), cholangiocyte marker surrounding luminal space (CK7), stellate cell marker (aSMA) and endothelial structures (CD31). Scale bars are 200 µm unless stated otherwise. (b) Wholemount immunostaining showing expression of various markers of liver cell types found within the organoids derived from hESC line H1. Showing hepatocyte markers (HNF4a and ASGR1), macrophage marker (CD68), cholangiocyte marker surrounding luminal space (CK7), stellate cell marker (aSMA) and endothelial structures (CD31). Scale bars are 200 µm unless stated otherwise. (c) Whole-mount live imaging of Oleic acid treated (right two panels) and untreated (left two panels) 207 derived organoids showing accumulation of non-polar fats after treatment (BODIPY in green). Oleic acid was used at 300 µM for 5 days from day 22. Scale bars are 200 µm unless stated otherwise. (d) Whole-mount live imaging of Oleic acid treated (right two panels) and untreated (left two panels) H1 derived organoids showing accumulation of non-polar fats after treatment (BODIPY in green). Oleic acid was used at 300 µM for 5 days from day 22. Scale bars are 200 µm unless stated otherwise.

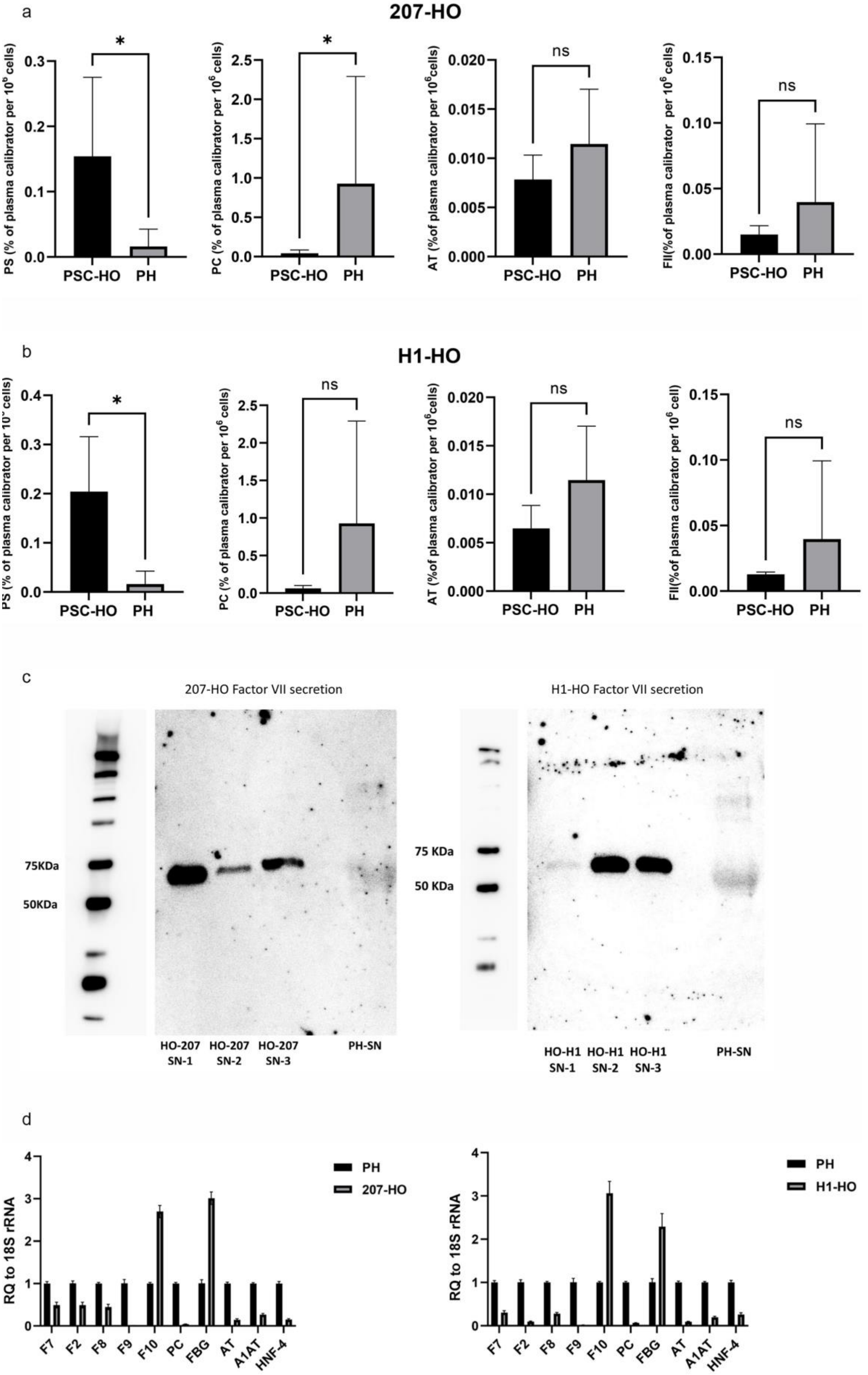
a
207-HO
PS (% of plasma calibrator per 10^6 cells)
PC (% of plasma calibrator per 10^6 cells)
AT (%of plasma calibrator per 10^6cells)
FII(%of plasma calibrator per 10^6 cells)
*
ns
PSC-HO
PH
b
H1-HO
FII(%of plasma calibrator per 10^6 cell)
c
207-HO Factor VII secretion
H1-HO Factor VII secretion
75KDa
50KDa
75 KDa
50 KDa
HO-207 SN-1
HO-207 SN-2
HO-207 SN-3
PH-SN
HO-H1 SN-1
HO-H1 SN-2
HO-H1 SN-3
PH-SN
d
RQ to 18S rRNA
PH
207-HO
H1-HO
F7
F2
F8
F9
F10
PC
FBG
AT
A1AT
HNF-4

Supplementary Figure 8. Characterising hESC (H1 and 207) derived organoids. (a and b) Analysis of hESC (207 and H1) derived organoids (PSC-HO), for production and secretion of the coagulation factor II (FII) and coagulation factor inhibitors PS, PC and AT measured using the Procarta-plex assay. The total concentration of FII, PS, PC and AT (black bars) were adjusted to $1x10^6$ cells and the results are expressed as the percentage of plasma calibrator control. Primary hepatocytes (PH, grey bars) were used as control. Results are from three independent experiments and are presented as mean ±SD. Statistical significance was assessed with Mann-Whitney test (* $p<0.05$). (c) Western assessment of hESC (207 and H1) derived organoids (HO) for the secretion of coagulation FVII into the culture medium. Primary hepatocytes (PH) were used as control. Three independent experiments using hESC (207 and H1) derived organoids (HO) were used. (d) Analysis of mRNA expression demonstrating levels of coagulation factors and inhibitors in hESC (207 and H1) derived organoids (grey bars) and primary human hepatocytes (black bars-PH). mRNA levels of coagulation factors II, VII, VIII, IX, X, fibrinogen (F2, F7, F8, F9, F10, FBG), coagulation inhibitors protein C and antithrombin (PC, AT) and the hepatic markers alpha-1 antitrypsin (A1AT) and hepatocyte nuclear factor 4 alpha (HNF4α) were determined using quantitative RT-qPCR with 18S as endogenous control. The results are presented as the mean of the fold-change expression of the respective gene. Results from three independent experiments are presented as mean ±SD.

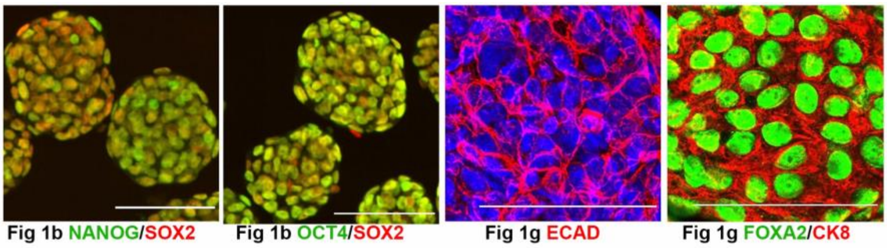

Fig 1b NANOG/SOX2 Fig 1b OCT4/SOX2 Fig 1g ECAD Fig 1g FOXA2/CK8

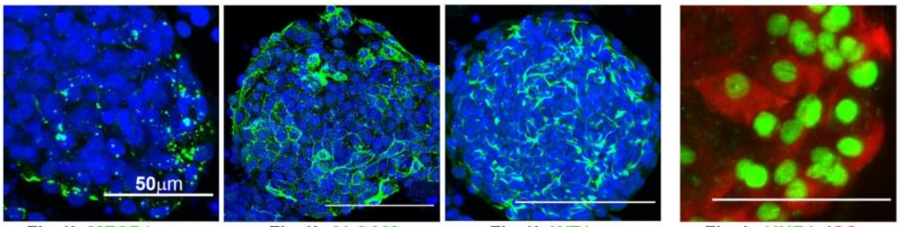

Fig 1h MESP1 Fig 1h ALCAM Fig 1h WT1 Fig 4a HNF4a/GS

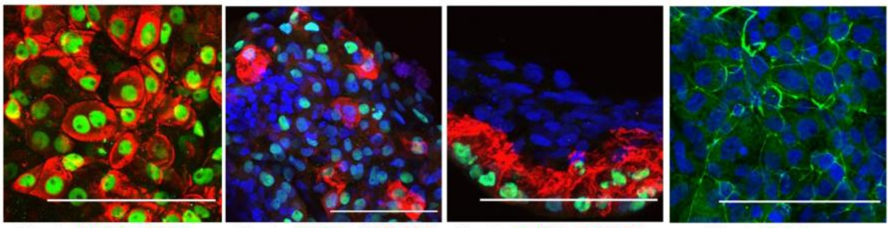

Fig 4a HNF4a/CPS1 Fig 4a HNF4a/CYP2A6 Fig 4a HNF4a/ASGR1 Fig 4a ZO-1

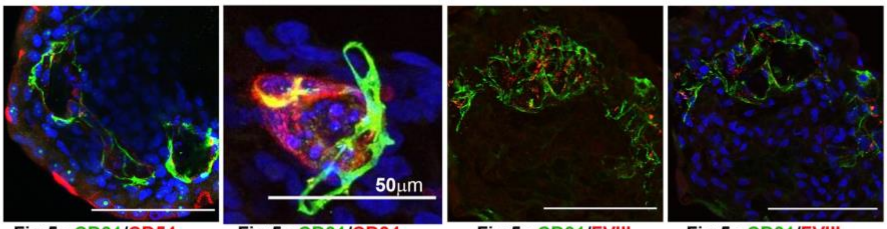

Fig 5a CD31/CD54 Fig 5c CD31/CD34 Fig 5e CD31/FVIII Fig 5e CD31/FVIII

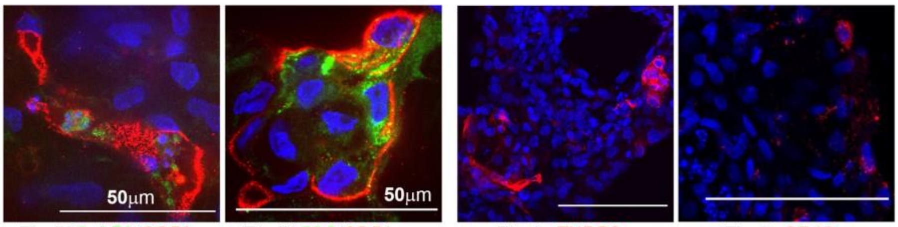

Fig 5f AcLDL/CD54 Fig 5f FSA/CD54 Fig 6a TUBB3 Fig 6c CD68

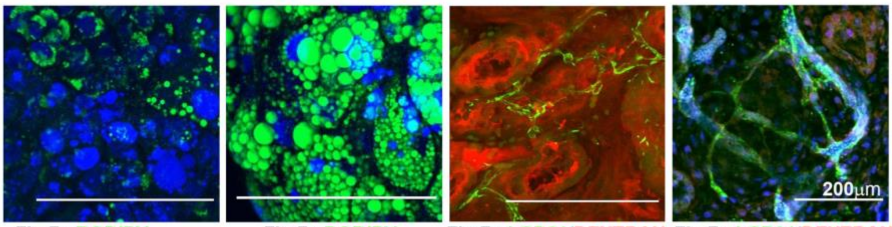

Fig 7e BODIPY Fig 7e BODIPY Fig 7g hCD31/DEXTRAN Fig 7g hCD31/DEXTRAN

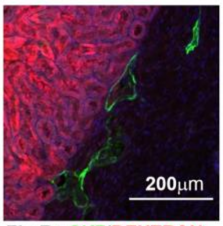

Fig 7g CK7/DEXTRAN

Supplementary Figure 9. Overview of zoomed images from the main manuscript. Zoomed in images from selected main figures (Fig. 1, 4, 5, 6, and 7) as indicated, to better visualise detail. Scale bars are 100 µm unless stated otherwise.

Supplementary Table 1. Overlapping and exclusive protein list linked to Fig 3a.

Supplementary Table 2

Taqman assay	Catalog number	Supplier
Hs01551992_m1 (F7)	4331182	Thermo Fisher Scientific
Hs01011988_m1 (F2)	4331182	Thermo Fisher Scientific
Hs00252034_m1 (F8)	4331182	Thermo Fisher Scientific
Hs01592597_m1 (F9)	4331182	Thermo Fisher Scientific
Hs00173450_m1 (F10)	4331182	Thermo Fisher Scientific
Hs00165584_m1 (PROC)	4331182	Thermo Fisher Scientific
Hs00170586_m1 (FGB)	4331182	Thermo Fisher Scientific
Hs00166654_m1 (SERPINC1)	4331182	Thermo Fisher Scientific
Hs01097800_m1 (SERPINA1)	4331182	Thermo Fisher Scientific
Hs00230853_m1 (HNF4A)	4331182	Thermo Fisher Scientific
Hs99999901_s1 (18S)	4331182	Thermo Fisher Scientific
Hs01005019_m1 (ASGR1)	4331182	Thermo Fisher Scientific
Hs01053049_s1 (SOX2)	444892	Thermo Fisher Scientific
Hs04260366_g1 (NANOG)	444892	Thermo Fisher Scientific
Hs00430824_g1 (MIXL1)	444892	Thermo Fisher Scientific
Hs00232764_m1 (FOXA2)	444892	Thermo Fisher Scientific
Hs00751752_s1 (SOX17)	444892	Thermo Fisher Scientific
Hs00242160_m1 (HHEX)	444892	Thermo Fisher Scientific
Hs00173490_m1 (AFP)	444892	Thermo Fisher Scientific
Hs00269972_s1 (CEBPA)	444892	Thermo Fisher Scientific
Hs00171403_m1 (GATA4)	444892	Thermo Fisher Scientific
Hs00426361_m1 (CYP3A7)	444892	Thermo Fisher Scientific
Hs00910225_m1 (ALB)	444892	Thermo Fisher Scientific
Hs00199611_m1 (TDO2)	444892	Thermo Fisher Scientific
Hs00610080_m1 (T)	4331182	Thermo Fisher Scientific
Hs00896293_m1 (PROX1)	4331182	Thermo Fisher Scientific
Hs00195612_m1 (TBX3)	4331182	Thermo Fisher Scientific

Hs00999634_gH (POU5F1)	4331182	Thermo Fisher Scientific
Hs00906630_g1 (GSC)	4331182	Thermo Fisher Scientific
Hs00415443_m1 (NODAL)	4331182	Thermo Fisher Scientific
Hs00193796_m1 (CER1)	4331182	Thermo Fisher Scientific
Hs00846731_s1 (SOX7)	4331182	Thermo Fisher Scientific
Hs00174914_m1 (TTR)	4331182	Thermo Fisher Scientific
Hs00952079_g1 (APOA2)	4331182	Thermo Fisher Scientific
Hs00604506_m1 (CYP3A4)	4331182	Thermo Fisher Scientific

Primary Antibodies, Secondary Antibodies, ELISAs and Serum

**Marker**	**Supplier**	**Catalogue No.**	**Species**	**Dilution**
αSMA	Invitrogen	MA5-11547	Rabbit	1:400
ALB	Sigma Aldrich	A6684	Mouse	1:600
ALB	Thermofisher	MA5-29022	Rabbit	1:100
ALCAM	Santa Cruz	sc-74558	Mouse	1:200
AFP	Sigma Aldrich	A8452	Mouse	1:600
ASGPR1	Santa Cruz	sc-5623	Mouse	1:200
B3 Tubulin	Santa Cruz	sc-80005	Mouse	1:200
Beta-actin	Sigma Aldrich	A5441	Mouse	1:5000
CD31	Abcam	ab32457	Rabbit	1:1000
CD34	Santa Cruz	sc-7324	Mouse	1:200
CD54	Thermofisher	MA5-1301	Mouse	1:100
CD68	Santa Cruz	sc-20060	Mouse	1:300
CD73	Sigma	HPA017357	Rabbit	1:500
CK19	Amersham	RPM1165	Mouse	1:150
CK7	DAKO	M7018	Mouse	1:100
CK7	Abcam	ab181598	Rabbit	1:100
CK8	DAKO	M0631	Mouse	1:100
CPS1	Santa Cruz	sc-376190	Mouse	1:150
CYP2A6	Origene	TA503832	Mouse	1:100
ECAD	Santa Cruz	sc-2179	Mouse	1:75
FII	Novus Biologicals	NBP1-58268	Rabbit	3μg/ml
FOXA2	Abcam	Ab108422	Rabbit	1:300
GS	Transduction Laboratories	G45020/L1	Mouse	1:250
HNF4α	Santa Cruz	sc-8987	Rabbit	1:300
HNF4α	Abcam	ab201460	Rabbit	1:100
LAM	Thermofisher	PA5-16287	Rabbit	1:250
LYVE1	Abcam	ab33682	Rabbit	1:200
MESP1	Thermofisher	PA5-67086	Rabbit	1:300
MRP2	Abcam	ab3373	Mouse	1:100
Nanog	Stemgent	09-0020	Rabbit	1:100
OCT4	Stemgent	09-0023	Rabbit	1:100
SOX2	Stemgent	09-0024	Rabbit	1:100
WT1	Thermofisher	PA5-16879	Rabbit	1:300
ZO1	Santa Cruz	sc-33725	Rat	1:50

Alexaflour'488'anti-rabbit	Life Technologies	A21206	Donkey	1:1500
Alexafluor'488'anti-mouse	Life Technologies	A11059	Rabbit	1:1500
Alexafluor'594'anti-mouse	Life Technologies	A11005	Goat	1:1500
Alexafluor'488'anti-rat	Life Technologies	A11006	Goat	1:1500
Mouse anti rabbit (HRP)	Santa Cruz	sc-2357		1:10000
m-IgGκ BP-HRP	Santa Cruz	sc-516102		1:10000
Phalloidin-594	Thermofisher	A12381		
Human Albumin ELISA	Bethyl Labs	e88-129		
Goat serum	Sigma	G9023		
